# Synthesis and evaluation of 14β-acyl substituted 17-cyclopropylmethyl-7,8-dihydromorphinone derivatives: mixed partial agonists at mu opioid and nociception/orphanin FQ peptide receptors

**DOI:** 10.1039/d5md00685f

**Published:** 2026-02-12

**Authors:** Mehrnoosh Ostovar, Keith Olsen, Gerta Cami-Kobeci, John R. Traynor, Luka Jeramaz, Stewart B. Kirton, Stephen M. Husbands

**Affiliations:** a Medicinal Chemistry Section, Department of Life Sciences, University of Bath Bath BA2 7AY UK s.m.husbands@bath.ac.uk; b Department of Pharmacology, and Edward F Domino Research Center, University of Michigan Ann Arbor MI 48109 USA; c Department of Medicinal Chemistry, University of Michigan Ann Arbor MI 48109 USA; d School of Health, Medicine and Life Sciences, University of Hertfordshire Hatfield Herts AL10 9AB UK; e School of Applied and Health Science, London South Bank University London SE1 0AA UK

## Abstract

Opioids remain the standard of care for management of severe pain, but adverse effects limit their use, particularly for the treatment of chronic pain. Compounds that have dual partial agonist activity at mu opioid (MOP) and nociceptin opioid peptide (NOP) receptors have been shown, in non-human primates, to display excellent analgesic activity with greatly reduced adverse effect profile. In this study we looked to increase the range of MOP/NOP dual acting compounds and, in particular, provide ligands with a greater diversity of MOP:NOP profiles. Reduction of the C_6_ carbonyl in the naltrexone scaffold to methylene resulted in a balanced MOP:NOP receptor profile in this series, in particular increasing potency at the NOP receptor. Ultimately, this will allow us to determine the optimal profiles for a range of therapeutic indications including pain and drug use disorders.

## Abstract 2

The development of a new series of mixed mu/nociceptin opioid partial agonists as analgesics with a greatly improved safety profile.

## Graphical abstract

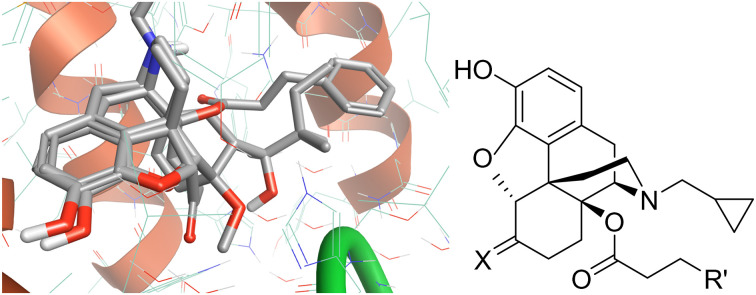

## Introduction

Mu opioid (MOP) receptor agonists remain a mainstay of treatment for pain, particularly associated with post-operative care, cancer and orthopaedic pain. While undoubtedly effective when used appropriately, current standard of care opioids cause a range of significant side effects that put limitations on their use. In particular, they have abuse-liability, cause respiratory depression and constipation, and suffer from the development of tolerance (so that greater doses are required over time to remain efficacious). The current opioid epidemic is partly due to chronic pain sufferers transitioning from appropriate use of prescription opioids to their misuse and abuse and illustrates the need to develop new, strong analgesics with a greatly improved side effect profile.

With these issues in mind, there has recently been interest in the development of opioids with mixed, partial agonist activity at the MOP and nociceptin opioid peptide (NOP) receptors. These compounds display strong analgesic activity in primate models of analgesia.^1–4^ Importantly, the lead compounds show significantly reduced abuse liability and physical dependence compared to standard of care opioids and do not cause respiratory depression, pruritus or constipation.

**Chart 1:**
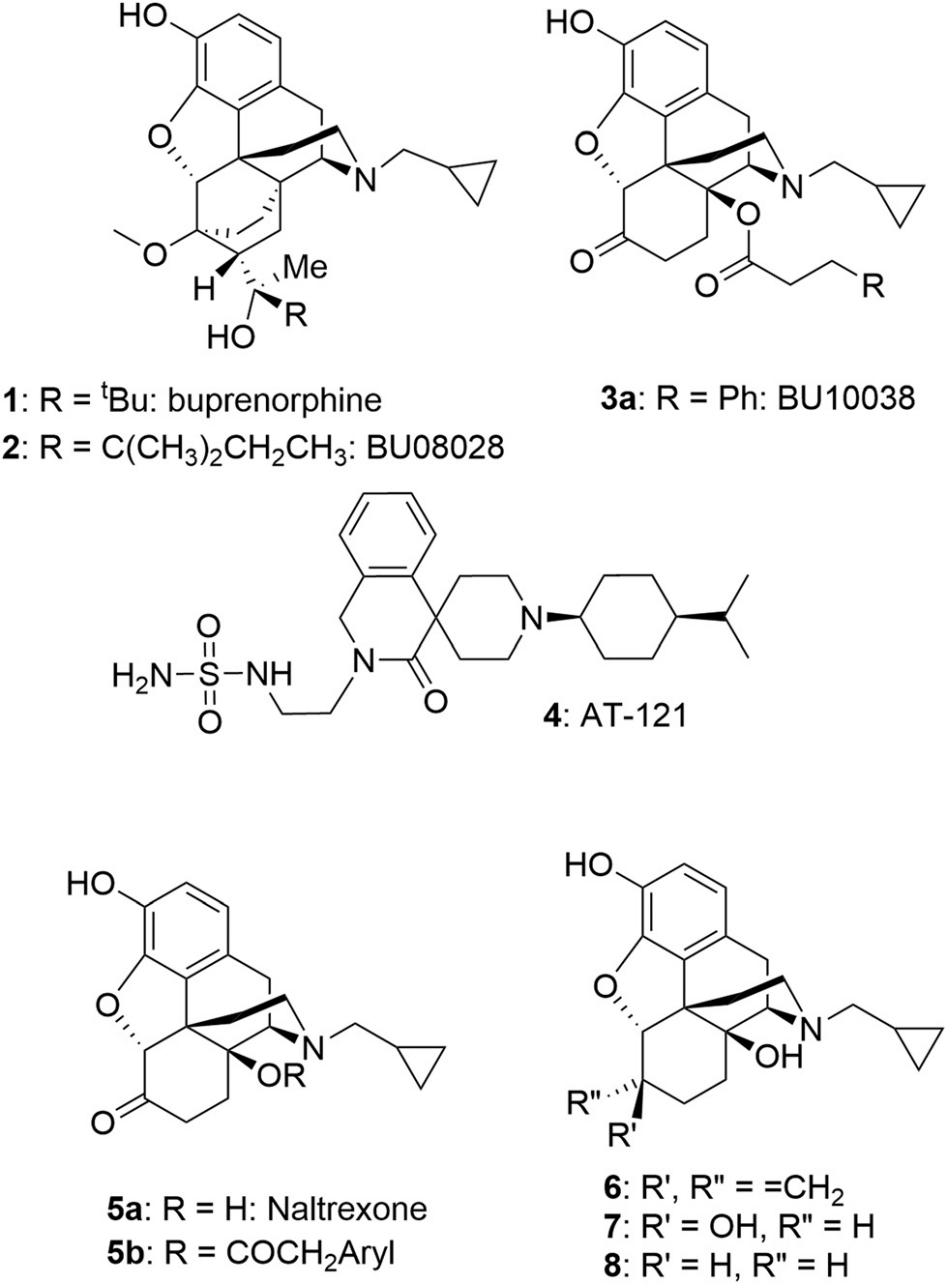
Several mu opioid and mixed mu/nociceptin opioid ligands.

Initially, the close buprenorphine (1) analogue BU08028 (2) (Chart 1) was developed, displaying the desired increased NOP activity relative to buprenorphine, but equivalent pharmacology to buprenorphine at MOP, kappa (KOP) and delta (DOP) receptors.^1,5^ Key findings were potent, long-lived analgesia in primates and the lack of side effects normally associated with MOP receptor mediated analgesia. Thus, in primates BU08028 had no effects on respiration, did not induce itching and had a greatly improved abuse liability profile (very limited self-administration) and no effect on respiration. To confirm that this promising profile was not molecule specific, compounds from different chemical series, but with equivalent pharmacology were sought. This led to BU10038 (3a) which helped confirm that these startling effects were a result of the MOP/NOP partial agonist activity. As with 2, 3a showed excellent analgesic properties of long duration and greatly reduced levels of self-administration compared to oxycodone (predicting low/no abuse liability). It also showed no tolerance after repeated dosing and had no effects on physiological signs such as respiration even at doses 10–30 times higher than for analgesia.^3^ At the same time Zaveri and colleagues developed AT-121 (4), again having dual partial agonist activity and a structure distinct from that of 2 and 3a. 4 again profiled as a strong analgesic with improved side-effect profile.^2^

While this appears to be a promising strategy for the development of new analgesics, it is not clear if the ideal balance between MOP and NOP activities has been achieved to ensure maximal analgesic activity whilst minimising the undesirable activities. While 2 and 3a have somewhat higher affinity for MOP compared to NOP receptors, the opposite is true for 4. To this end, we now report on a series of close analogues of 3a, where an increase in relative NOP activity was sought.

### Design

In the series of phenacetyl esters (5b) of naltrexone (5a), closely related to 3a, substitution of the side chain aryl ring had a marginal effect on affinity and efficacy for NOP but did modulate efficacy at MOP and KOP receptors.^6^ As the aim of the current study was to increase NOP affinity but leave MOP and KOP affinity and efficacy unchanged (and certainly not raised), rather than exploring ring-substitution of 3a, we looked to alternative ring systems (specifically, heterocyclic rings) as one approach and, more interestingly, the importance of the C_6_ keto group. It is well known that the carbonyl group of 5a can be subtly modified to a methylene (6: nalmefene), a hydroxy group (7: 6β-naltrexol) or removed (8: 6-deoxynaltrexone) without significantly affecting affinity for MOP receptors. As 6 has partial agonist activity at KOP receptors^7^ and 7 has reduced ability to enter the CNS,^8^ our focus was on removal of the carbonyl as 8 retains high affinity and very low/no efficacy at MOP and KOP receptors and should retain CNS access.^9^

## Results

### Synthetic procedures

**Scheme 1:**
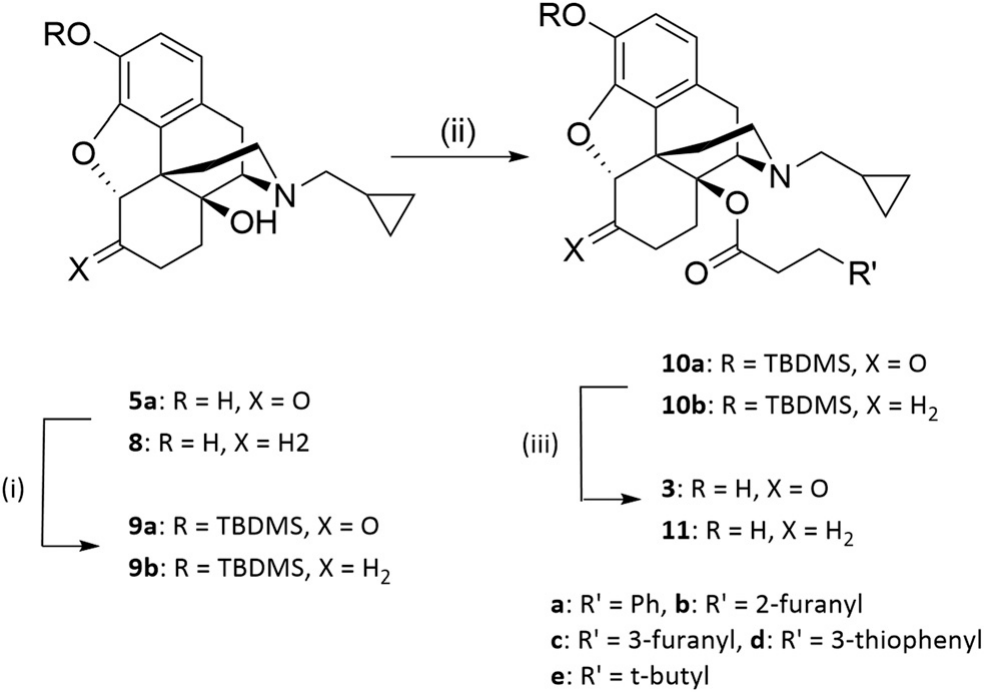
Synthesis of 14-*O*-acylated naltrexone and 6-deoxynaltrexone analogues. Reagents and conditions: i) TBDMSCl, imidazole, DCM, rt ii) (R′CH_2_CH_2_CO)_2_O, toluene, 125 °C iii) TBAF, THF, rt.

6-Deoxynaltrexone (8) was prepared using standard Wolff–Kishner conditions of hydrazine and KOH, as described previously.^9^ Introduction of the side chain was as previously reported for the phenacetyl analogues (Scheme 1).^6^ The 3-hydroxy group of 5a and 8 was protected with *tert*-butyldimethylsilyl chloride using a standard method^10^ followed by esterification of the 14-hydroxy group. The tendency of the C_6_-carbonyl to exist in its enol form meant that cleaner esterification was achieved with the appropriate anhydrides rather than with acyl chlorides. The anhydrides were synthesized from the corresponding phenylacetic acid and triphosgene.^11^ Thereafter the 3-hydroxy group was regenerated using a 1 : 1 mixture of methanol and HCl (6N) to give the target esters (3 and 11). (Scheme 1).

### Pharmacological evaluation

Each analogue was tested for binding affinity in DOP-, KOP-, MOP-, and NOP receptors heterologously expressed in Chinese hamster ovary (CHO) cells (Table 1), followed by functional analysis in the [^35^S]GTPγS assay in the same cell lines (Table 2). All compounds retained high affinity at MOP, DOP and KOP receptors. Most of the new compounds showed increased NOP affinity and reduced MOR efficacy relative to buprenorphine (Table 1). 11a, 3a (the latter previously reported^4^) and 11d showed the highest NOP affinity and potency (Tables 1 and 2). No significant degree of agonist activity (stimulation <15% compared to standards) was observed at KOP or DOP receptors.

**Table 1:** Binding affinities at NOP, MOP, KOP and DOP receptors

	R	hNOP	hMOP	hKOP	hDOP
		*K*_i_ (SEM)/nM	*K*_i_ (SEM)/nM	*K*_i_ (SEM)/nM	*K*_i_ (SEM)/nM
Naloxone			10 (1.4)	0.6 (0.2)	46 (9.9)
Nociceptin		0.030 (0.006)			
Buprenorphine 1		1300 (321)	1.3 (0.17)	0.87 (0.43)	10 (2.6)
Naltrexone 5a		>10 000	1.2 (0.15)	3.7 (1.1)	53 (12)
3a	Phenyl	177 (40)	0.16 (0.08)	0.79 (0.40)	0.42 (0.04)
3b	2-Furanyl	229 (58)	1.0 (0.67)	0.35 (0.12)	4.5 (1.2)
3c	3-Furanyl	352 (67)	0.16 (0.09)	0.20 (0.14)	4.3 (1.7)
3d	3-Thiophenyl	240 (37)	0.17 (0.10)	0.14 (0.05)	1.5 (0.6)
3e	*t*-Butyl	>1000	0.77 (0.15)	0.17 (0.04)	13.5 (6.8)
11a	Phenyl	16 (5)	0.16 (0.11)	0.91 (0.69)	7.5 (0.18)
11b	2-Furanyl	120 (29)	0.0082 (0.0011)	0.019 (0.007)	0.0013 (0.004)
11c	3-Furanyl	127 (43)	0.0024 (0.0008)	0.035 (0.008)	0.0031 (0.001)
11d	3-Thiophenyl	35 (10)	0.0068 (0.0022)	0.007 (0.002)	0.0044(0.0024)
11e	*t*-Butyl	563 (110)	0.0067 (0.0007)	0.032 (0.021)	0.034 (0.14)

**Table 2:** Functional activity in the [^35^S]GTPγS assay at NOP, MOP, KOP and DOP receptors

	R	hNOP		hMOP		hKOP	hDOP
		EC_50_ (SEM)/nM	*E*_MAX_ (SEM)	EC_50_ (SEM)/nM	*E*_MAX_ (SEM)	*E*_MAX_ (SEM)	*E*_MAX_ (SEM)
DAMGO				33 (3.1)	100 (5.0)		
Nociceptin		0.52 (0.061)	98 (3.7)				
Buprenorphine^a^		>1000	35 (3.8)	0.79 (0.31)	34 (6.0)	<15% stim	<15% stim
Naltrexone		NS		5.5 (3.8)	−8 (3)	<15% stim	<15% stim
3a^a^	Phenyl	160 (51)	47 (9.0)	1.5 (0.67)	28 (4.4)	<15% stim	<15% stim
3b	2-Furanyl	270 (79)	42 (7.9)	0.20 (0.044)	24 (3.8)	<15% stim	<15% stim
3c	3-Furanyl	286 (25)	34 (3.3)	0.18 (0.054)	18 (1.3)	<15% stim	<15% stim
3d	3-Thiophenyl	174 (82)	49 (5.0)	0.041 (0.019)	22 (3.8)	<15% stim	<15% stim
3e	*t*-Butyl	472 (116)	24 (4.4)	0.23 (0.14)	23 (4.4)	<15% stim	<15% stim
11a	Phenyl	0.97 (0.22)	47 (11)	3.9 (1.3)	23 (7.1)	<15% stim	<15% stim
11b	2-Furanyl	50 (7.6)	35 (6.9)	1.50 (0.80)	28 (2.2)	<15% stim	<15% stim
11c	3-Furanyl	49 (12)	31 (5.0)	0.88 (0.012)	23 (4.4)	<15% stim	<15% stim
11d	3-Thiophenyl	2.6 (0.64)	48 (3.3)	0.064 (0.020)	26 (0.29)	<15% stim	<15% stim
11e	*t*-Butyl	359 (135)	30 (7.0)	0.79 (0.24)	26 (0.29)	<15% stim	<15% stim

a Taken from ref. 4.

The majority of compounds had higher affinity for each of the three classical opioid receptors than naltrexone. This was observed in the 6-keto series (3) where there was an approximate 10-fold increase across the receptors but was most striking for the heterocyclic 6-deoxy series (11) where affinities for all 3 classical opioid receptors were in the pM range. At NOP, where naltrexone had no measurable affinity, all but the *t*-butyl substituted compounds 3e and 11e, had good affinity, again with the 6-deoxy series showing higher affinity than their 6-keto counterparts, that was substantially higher (5–10-fold) than buprenorphine's for the same receptor. The clearest SAR related to removal of the carbonyl group, which led, in almost every case, to a substantial increase in affinity over the 6-keto equivalent for each receptor. The exception to this finding was 11a, where the only rise in affinity was at NOP receptors, leading to a more balanced MOP/NOP affinity profile than with any of the other compounds.

In functional assays the compounds profiled as MOP receptor partial agonists with efficacies similar to, or lower than, buprenorphine. All the new compounds were also partial agonists at NOP receptors and substantially more potent than buprenorphine. As predicted by their binding affinities, the 6-deoxy series (11) were more potent at the NOP receptor than their 6-keto counterparts; however, this was not the case at MOP receptors, where broadly similar potencies were found across the two series. The 6-deoxy compounds 11a (phenyl substituted) and 11d (3-thiophenyl substituted) stood out for their high potency at NOP receptors and the comparative (to other compounds in the series) low potency of 11a at MOP receptors meant that it profiled as a more balanced mixed MOP/NOP receptor partial agonist (4 nM *versus* 1 nM), approximately 100-fold more potent at NOPr than its keto counterpart 3a. Indeed, 11a was more potent at NOP than MOP receptors. Importantly, all compounds retained very low efficacy at KOP and DOP, so that the series has the desired MOP/NOP partial agonist, KOP/DOP antagonist profile.

### *In silico* studies

Calculated log *P* values were obtained from a number of widely available *in silico* tools *i.e.* Flare (v7.2.0, Cresset), virtual log *P*,^20^ Molinspiration,^21^ MOE (v2022.02),^22^ SwissADME^23,28^ and VCCLab^24^ (Table 3). Using a variety of validated tools reduces the potential for bias in any single algorithm and allows for more robust consideration of any trends observed. As expected, the reduction of the C_6_ from a carbonyl (series 3) to methylene (series 11) resulted in an increase in hydrophobicity. This is demonstrated for each of the algorithms included in the study, albeit the difference is less pronounced with MOE and VCCLab when compared to the others. Within series the furan derivatives on average were substantially less lipophilic than their thiophenyl, phenyl and *t*-butyl analogues, but the absolute difference was less pronounced for the virtual log *P* algorithm when compared to the others.

**Table 3:** Calculated log *P* values

Ligand	Flare	Virtual log *P*	Mol-inspiration	MOE	Swiss-ADME	VCCLab	Mean	STDEV	Relative rank
3a (phenyl)	3.7	3.593	3.86	*3.02*	3.64	**4**	3.64	0.3	8
3b (2-furanyl)	2.7	**3.595**	2.87	*2.37*	2.99	3.28	2.97	0.4	9
3c (3-furanyl)	2.7	**3.507**	2.8	*2.37*	2.98	3.34	2.95	0.4	10
3d (3-thiophenyl)	3.8	4.004	3.44	*2.99*	3.69	**4.06**	3.66	0.4	6
3e (*t*-butyl)	*3.9*	**4.55**	4.28	4	4.07	4.17	4.16	0.2	4
11a (phenyl)	4.5	4.484	**4.99**	*3.22*	4.38	4.48	4.34	0.6	3
11b (2-furanyl)	3.5	**4.529**	4	*2.56*	3.73	3.76	3.68	0.7	5
11c (3-furanyl)	3.5	**4.437**	3.93	*2.56*	3.72	3.75	3.65	0.6	7
11d (3-thiophenyl)	4.6	**4.904**	4.58	*3.18*	4.37	4.61	4.37	0.6	2
11e (*t*-butyl)	4.7	5.298	**5.41**	*4.2*	4.71	5.24	4.93	0.5	1

The variations in NOP affinity (*K*_i_) and potency (EC_50_) broadly followed the changes in lipophilicity – this was particularly noticeable within the 6-deoxo series 11. Thus, in each case, the Ph and thiophenyl analogs have greater potency than their furanyl counterparts. The exceptions to this, in both series, were the *t*-Bu containing analogs 3e and 11e, which both display highest lipophilicity but lowest potency within their series.

Absolute correlations between affinity and lipophilicity are difficult to ascertain,^29,30^ but it is often useful to use comparisons of relative rankings (*i.e.* Spearman's rankings) to determine whether general trends in lipophilicity are correlated to biological activities. Although this is not a perfect comparison by any means, as it assumes uniform distribution between the endpoints of each range of the data series concerned, it can highlight trends and provide a testable hypothesis such as the more lipophilic a compound the higher the biological affinity.

To this end, the mean calculated log *P* for each compound was ranked from 1 (most lipophilic) to 10 (least lipophilic). Similarly, the experimentally observed biological activities for each of the compounds against each of the opioid receptors were ranked from 1 (most active) to 10 (least active). The relative rank of mean lipophilicity *versus* relative rank for activity against each isoform (NOP, MOP, KOP and DOP) was plotted and an *r*^2^ value calculated (Table 4).

**Table 4:** Relative ranking of mean log *P vs.* activity against hXOP isoforms

Ligand	Relative rank mean log *P*	Relative rank hNOP affinity	Relative rank hMOP affinity	Relative rank hKOP affinity	Relative rank hDOP affinity
3a (phenyl)	8	3.7	3.593	3.86	3.02
3b (2-furanyl)	9	2.7	3.595	2.87	2.37
3c (3-furanyl)	10	2.7	3.507	2.8	2.37
3d (3-thiophenyl)	6	3.8	4.004	3.44	2.99
3e (*t*-butyl)	4	3.9	4.55	4.28	4
11a (phenyl)	3	4.5	4.484	4.99	3.22
11b (2-furanyl)	5	3.5	4.529	4	2.56
11c (3-furanyl)	7	3.5	4.437	3.93	2.56
11d (3-thiophenyl)	2	4.6	4.904	4.58	3.18
11e (*t*-butyl)	1	4.7	5.298	5.41	4.2
*R* ^2^ values	—	0.023	0.2292	0.2178	0.0194

Pairwise comparisons of relative ranks showed that there was no correlation between relative lipophilicity and relative affinity rankings across the dataset as a whole with *r*^2^ values ranging from 0.023 to 0.23.

It was possible that this analysis could have been skewed by variations in individual log *P* calculation algorithms. To this end pairwise Spearman rank correlation between rankings for each algorithm were carried out to identify any models that were potentially skewing the data. This showed that there was no significant variation between relative rankings for any of the algorithms at 95% confidence, and hence a general observation that lipophilicity is a proxy measure for biological affinity does not hold true for this set of compounds.

To explore this further, experiments were conducted to determine if there was a stronger correlation between relative rankings for calculated aqueous solubility of the compounds and their relatively ranked binding affinities. The algorithms investigated were VCCLabs,^24^ the TPSA model,^25^ Molinspiration,^21^ SILICOSIT,^26^ SwissADME^27^ and the log *S* calculation in MOE.^22^ Pairwise Spearman ranking showed correlation between the relative rankings at approximately 95% confidence for ESOL and the hMOP binding affinities (*α* = 0.054) and ESOL and the hDOP binding affinities (*α* = 0.054) but no correlation between ESOL and the other two isoforms hKOP and hNOP or any of the other solubilities generated by the remaining algorithms and the binding affinities for any isoform.

This suggests that affinity and potency is more nuanced than solely being a function of a holistic physicochemical property, and as such docking studies were conducted to investigate interactions between the molecules and the isoforms at the molecular level.

**Fig. 1:**
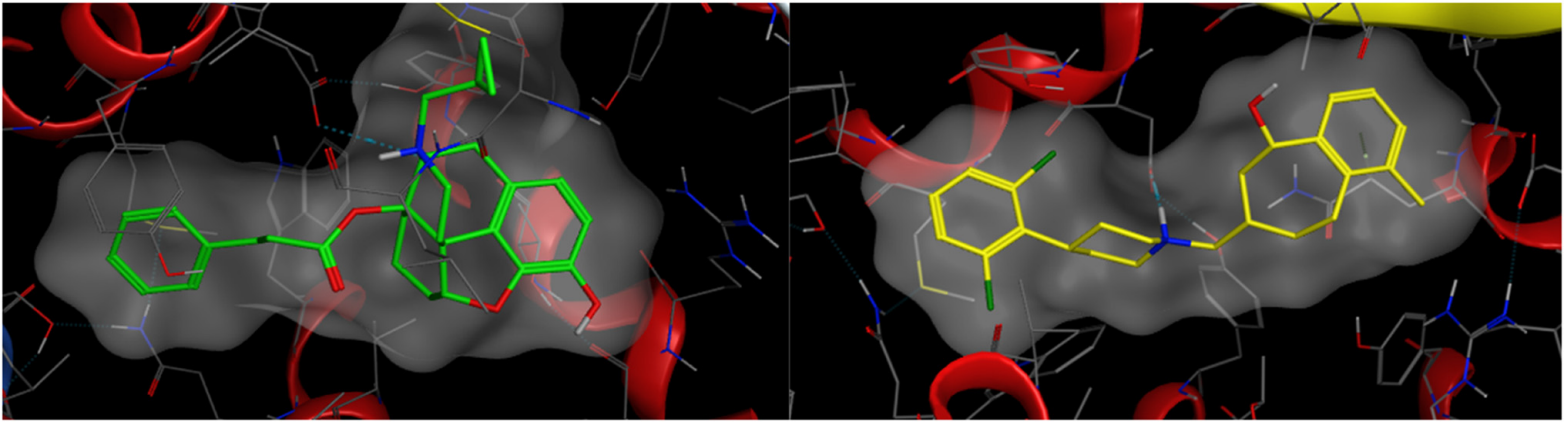
Left – Docking pose illustrated for 11a and Right – the equivalent crystal structure orientation of SB-612111 from PDB: 5DHH.

A comparison between PDB: 5DHH and an alternative NOP structure, PDB: 5DHG, showed that both had the same resolution (3.00 Å), both contained co-crystallized antagonist ligands, and had no Ramachandran outliers. Both structures contained mutations, but amino acid sequences were identical, and neither contained mutations in the binding pocket. As such either structure could be used. Another comparison was made, comparing 5DHH to 8F7X which represented the active state of NOP co-crystallized with nociceptin. A root mean square deviation (RMSD) of all atoms was taken and it revealed a structural difference of 1.37 Å. The volumes of 5DHH and 8F7X binding pockets were 932.9 Å^3^ and 1150 Å^3^ respectively. Using the sequence alignment tool in MOE, their sequences were found to be identical from 5DHH's residue 117 (glycine) to residue 414 (glycine) encompassing both of their binding pockets. MOE's “SiteView” feature demonstrates a key difference in their binding pockets where 8F7X contained more amino acid residues in its pocket (76) than 5DHH (59), consistent with the increased volume of the former. Ultimately, docking studies were performed using the inactive state of the NOP receptor (PDB: 5DHH), a choice justified by the low efficacy, partial agonist character of the ligands. The protein was downloaded from the RCSB Protein Data Bank and a full preparation of the protein–ligand complex performed on import into Flare. Docking (within Flare) used the ‘very accurate but slow’ docking mode in a region defined by the crystal structure ligand (SB-612111) and expanded to include protein atoms within 6 Å. A docking constraint was added so that the preferred binding modes allowed a salt bridge between the protonated N17 of the ligands and Asp130 of the protein active site. For comparison, docking was also carried out within MOE, again employing a docking constraint to favour binding poses with the salt bridge. Docking was then performed using the triangle matcher placement method and rigid receptor refinement with the binding site defined by the co-crystallized ligand. Flare and MOE produced very consistent binding poses with the C_14_ side chain phenyl ring extended into a region bounded by Tyr131^3.33^, Meth134^3.36^, Val283^6.55^, and Ile219^5.42^ amongst other residues (Fig. 1a) (superscript figures indicate Ballesteros Weinstein numbering). This is the region accessed by the dichlorophenyl ring of SB-612111 in the crystal structure (Fig. 1b). C_6_ of the new series of ligands projects towards Val279^6.51^, Tyr 309^7.43^, and Leu301^7.35^ in this pose.

**Fig. 2:**
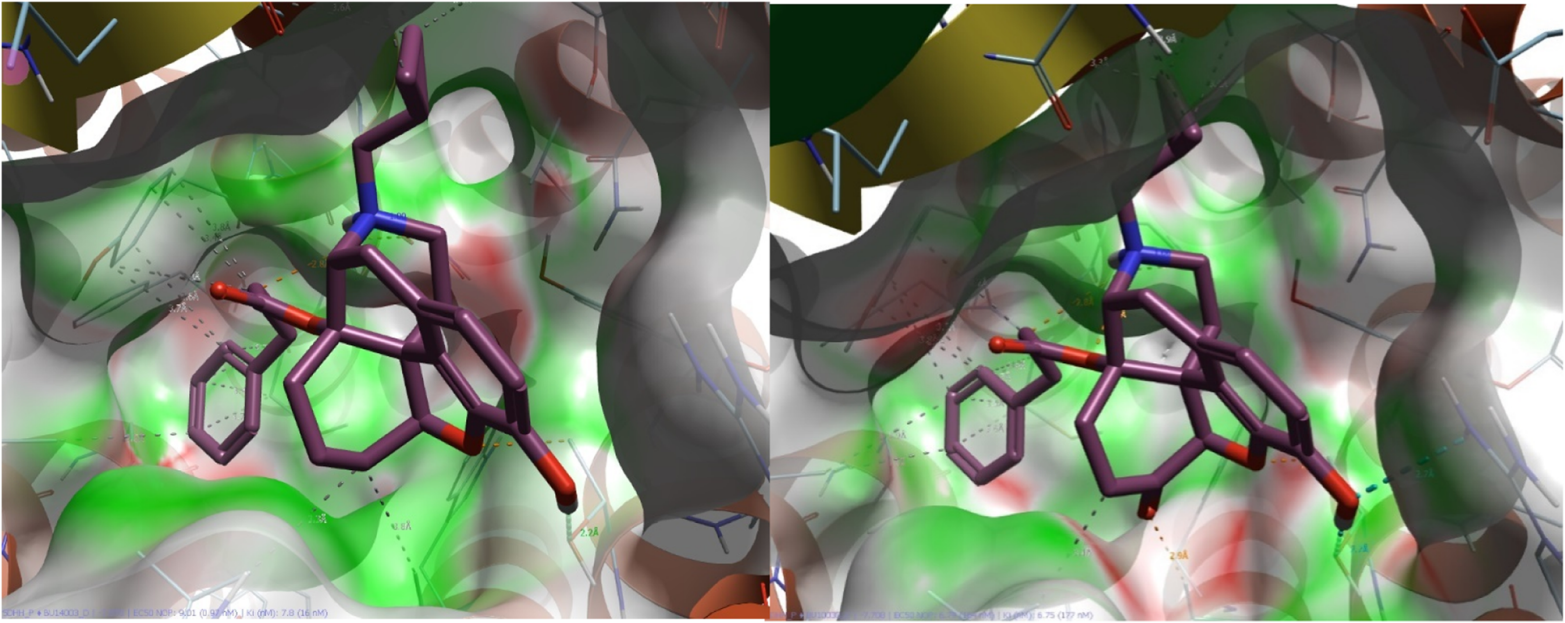
Surface coloured by complementarity between ligands electrostatic potential and the protein (green = good, red = bad). Left) Region around C_6_ of 11a is highly complementary to the NOP receptor protein and Right) equivalent pose for 3a indicates poor complementarity between C_6_ region and the protein.

Compounds 3a and 11a adopt closely related binding orientations, with both ligands positioning their shared aromatic scaffold in an area around Thr305, Asp130, Tyr309 amongst other residues and maintaining the same key N17 to Asp 130 salt bridge. The only structural distinction between the 3a and 11a is a carbonyl *versus* methylene at C_6_, but this does not alter the overall pose, and each ligand. In Fig. 2, surface complementarity between the NOP receptor protein and ligands 3a and 11a is displayed. Series 11 (6-deoxy) has improved electrostatic complementarity with the protein compared to the 6-keto analogues (series 3) but differences are small and do not account for the substantial differences in potency.

The interactions of the most potent ligand (11a), and the least potent ligand (3e) with the binding pocket were also compared. While both ligands were predicted to form a salt bridge between the protonated N17 and Asp130^3.32^, 11a was a better fit for the pocket with both the core scaffold and the side chain well-buried within the binding site, resulting in minimal solvent exposure. With 3e the side chain and part of the core structure extended outward into the solvent-exposed region resulting in more solvent exposure.

Since all the new ligands bound with good affinity to the MOP a comparison was made between β-FNA, the co-crystallised ligand in 4DKL and the new series 3 and 11. 11e has an 88.78% Tanimoto similarity to β-FNA which is the highest out of any ligand in series 11 and series 3. The RMSD of the poses was 1.79 Å. Both ligands had a salt bridge between the protonated N17 and Asp147^3.32^. As expected their core structures overlap with each other and they both project towards Val300^6.55^, Val236^5.42^, Lys233^5.39^ with just the side chains extending towards a different regions of the binding pocket (SI).

## Discussion

**Chart 2:**
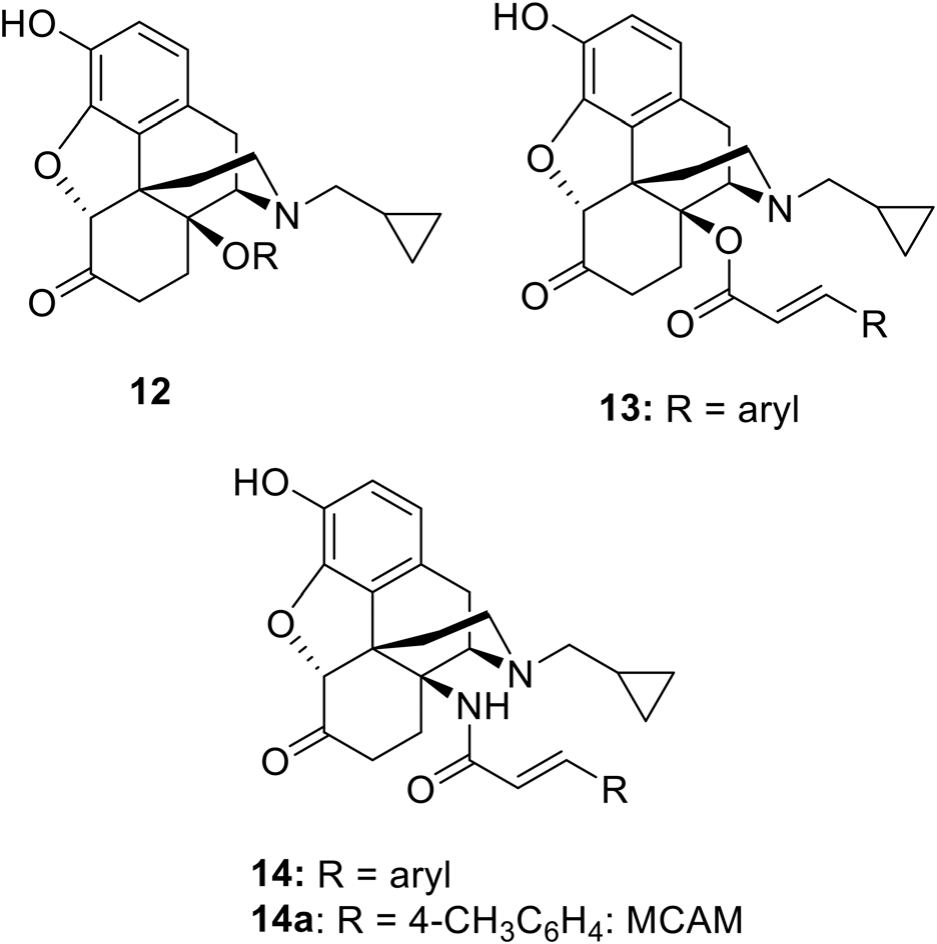
Related C_14_-substituted analogues of naltrexone.

C_14_ analogs and derivatives of naltrexone have provided some of the most interesting opioid series, with a wide range of pharmacological profiles achievable. 14-*O*-Alkyl ethers of naltrexone (12: Chart 2) tend to have predominant MOP receptor antagonist activity^12,13^ though the 14-*O*-3-phenylpropyl ether is a high efficacy and high potency MOP agonist.^14^ Cinnamoyl esters (13), which differ from the lead, 3a, only in having a double bond in the side chain, are predominantly opioid receptor antagonists.^15^ The amide equivalents of the cinnamoyl esters are the 14-*N*-cinnamoylamino derivatives (14). These are similarly potent antagonists but of longer duration, as exemplified by the MOP-receptor selective irreversible antagonist methocinnamox (M-CAM; 14a)^16–18^ currently in preclinical development with potential as a relapse prevention agent and for overdose reversal and/or prevention.^19^ Closely related to the current series are the 14β-phenylacetyl substituted analogues (5b: Chart 1).^6^ These compounds profiled as partial agonists across each of the four opioid receptors, with affinities and potencies at NOP receptors very similar to buprenorphine. The current series of esters having, as predicted, partial agonist activity at MOP receptors, fall in the middle of this range.

A key priority in the current study was to further increase affinity and agonist activity at NOP receptors. The lead, 3a, has a highly desirable profile in non-human primates with evidence of agonist activity at both MOP and NOP receptors.^3,4^ Our *in vitro* findings differ slightly from the original report on 3a. Compared to Kiguchi *et al.*^3^ we obtained a 10-fold lower affinity and a 4-fold lower potency in the [^35^S]GTPγS assay at the NOP receptor.^4^ However, the levels of agonist efficacy are similar as are the binding affinities at the KOP and DOP receptors; both sets of data show no agonist activity at KOP or DOP receptors.

Introduction of heteroaryl rings in place of the phenyl ring of 3a resulted in increased selectivity towards the MOP receptor, as did replacement of the aryl ring with a *t*-butyl group. On reducing the C_6_ carbonyl to methylene, binding affinities for all receptors tended to increase, whereas in the [^35^S]GTPγS assay only potency at the NOP receptor was significantly altered (increased), leading to compounds with a more balanced MOP:NOP receptor profile and, in 11a, the first compound in this series identified as having higher potency at NOP- than MOP receptors. It will be of interest to see how this alters the *in vivo* profile of the compound. The fact that 11a has a lower affinity for the NOP receptor than the MOP receptor but a higher potency may relate to the different assay conditions for the two measures. Binding assays were performed in Tris buffer whereas the [^35^S]GTPγS assay was performed in a buffer containing 125 mM NaCl and 5 mM MgCl_2_, as well as GDP.

The increase in lipophilicity on going from the keto to the deoxo series is unlikely to explain the increase in potency of the latter series as the more lipophilic members of the keto series (3d and 3a) are substantially less potent than all of the deoxo series despite having greater lipophilicity than 11b and 11c. Increased lipophilicity does increase the difference in NOP affinity and potency between the reduced series 11 and the carbonyl containing series 3. Thus, 11a is 169 times more potent than 3a, there is a 68-fold difference for the thiophenes and a 5–6 fold difference for the furans.

The variation in potency and efficacy at MOP and NOP receptors through the series will allow future *in vivo* work to determine the optimal balance of MOP and NOP activity for strong analgesia with minimal side effects. For example, 3a, 11d and 11a have virtually identical efficacies to each other at MOP and NOP, but selectivity in potency varies from ∼100-fold for MOP (3a), ∼55-fold for MOP (11d) to 4-fold for NOP (11a).

## Experimental

### Synthesis and characterization

Reagents and solvents were purchased from Sigma-Aldrich or Alfa Aesar and used as received. ^1^H and ^13^C NMR spectra were obtained with a Brucker-400 MHz instrument (^1^H at 400 MHz, ^13^C at 100 MHz); residual solvent resonances were used as internal reference signals. ESIMS: microTOF (BRUKER). Column chromatography was performed using pre-packed columns on a Teledyne ISCO combiflash instrument. Ligands were tested as their hydrochloride salts, prepared by adding 5 equivalents of HCl (1 N solution in diethyl ether) to a solution of compound in anhydrous methanol. All reactions were carried out under an inert atmosphere of nitrogen unless otherwise indicated. All compounds were >95% pure.

#### 3-*O*-TBDMS-naltrexone and 3-*O*-TBDMS-6-deoxynaltrexone (9a & 9b)

To a solution of 5a.HCl or 8.HCl (0.66 mmol) in DCM (12 mL), imidazole (157 mg, 2.31 mmol), and *tert*-butyl-dimethyl-silyl-chloride (TBDMSCl) (149 mg, 0.99 mmol) were added. The solution was stirred at room temperature for 18 h. 10 mL of water added at 0 °C and the aqueous layer extracted (3 × 15 mL) with DCM. The combined organic extracts were washed with 10% aqueous HCl (15 mL), following by water (15 mL) and brine (15 mL), dried over MgSO_4_ and evaporated to dryness. The resulting pale-yellow solid was purified by column chromatography over 12 g *R*_f_ silica column using *R*_f_ combi flash machine (30–50% ethyl acetate in hexane), *R*_f_ = 0.17 (at 30% EtOAc/hexane, 0.5% NH_3_), affording 9a & 9b as white solids (>85%).

#### General method for preparation of 3-*O*-TBDMS-14-propanoyl esters (10a, 10b)

To a stirred solution of 9a or 9b (0.87 mmol), toluene (9.0 mL), was added the appropriate anhydride (2.19 mmol), under nitrogen atmosphere. After 18 hours at reflux the reaction mixture was cooled to room temperature and treated with an excess of saturated aqueous NaHCO_3_, then extracted with DCM (×3). The extracts were washed with brine, dried over MgSO_4_ and evaporated to dryness. The resulting cream foam solid was purified by column chromatography over 24 g *R*_f_ silica column using *R*_f_ combi flash machine (30–50% ethyl acetate in hexane) and then used immediately in the next step.

#### De-protection of TBDMS group (3, 11)

To a stirred solution of 10a or 10b (0.5 mmol) in anhydrous THF (3 mL), was added solution of tetrabutyl ammonium fluoride 1.0 M in THF (708 μL, 0.78 mmol). The reaction mixture was stirred at room temperature for 1 hour. The mixture was treated with an excess of saturated aqueous NH_4_Cl and extracted with DCM (×3). The extracts were washed with brine, dried over MgSO_4_ and evaporated to dryness. The resulting cream foam solid was purified by column chromatography over 24 g *R*_f_ silica column using *R*_f_ combi flash machine (30–50% ethyl acetate in hexane, 0.5% NH_4_OH), affording 14-acylated materials as white solids.

##### 14β-(3-Phenylpropanoyl)-17-cyclopropylmethyl-7,8-dihydronoroxymorphinone (3a)

Isolated as a white solid (97%). *R*_f_ 0.32 (at 30% EtOAc/hexane, 0.5% NH_3_); ^1^H NMR (CDCl_3_) *δ* 0.03–0.08 (2H, m), 0.42–0.46 (2H, m), 0.64–0.73 (1H, m), 1.43–1.50 (1H, m), 1.51 (1H, dt, *J* = 3.72 Hz & 14.44 Hz), 2.09–2.18 (3H, m), 2.20–2.54 (5H, m), 2.63–2.86 (3H, m), 2.98–3.02 (2H, m), 3.05 (1H, d, *J* = 18.2 Hz), 4.43 (1H, d, *J* = 5.52 Hz), 4.58 (1H, s), 6.55 (1H, d, *J* = 8.0 Hz), 6.68 (1H, d, *J* = 8.0 Hz), 7.14–7.31 (5H, m); ^13^C NMR, 400 MHz, (CDCl_3_) *δ* 3.84, 9.51, 23.06, 26.91, 30.10, 31.05, 35.44, 36.61, 43.87, 51.24, 55.57, 59.46, 82.55, 90.16, 117.89, 119.97, 125.29, 126.42, 128.05, 128.22, 128.25, 128.49, 128.59, 129.02, 138.60, 140.49, 143.30, 171.80, 208.51. HRMS, *m*/*z* for (C_29_H_32_NO_5_) [MH]^+^, calcd – 474.2280, found – 474.2245. Anal (C_29_H_31_NO_5_·HCl·H_2_O) C, H, N.

##### 14β-(3-(Furan-2-yl)propanoyl)-17-cyclopropylmethyl-7,8-dihydronoroxymorphinone (3b)

The product, free base, was obtained as a white foam (80%). ^1^H NMR (400 MHz, CDCl_3_) *δ* 7.29–7.26 (m, 1H), 6.70 (d, *J* = 8.0 Hz, 1H), 6.55 (d, *J* = 8.0 Hz, 1H), 6.25 (dd, *J* = 2.0, 3.2 Hz, 1H), 6.06–6.03 (m, 1H), 4.62 (s, 1H), 4.42 (d, *J* = 5.5 Hz, 1H), 3.08–3.0 (m, 3H), 2.86–2.74 (m, 3H), 2.66 (dd, *J* = 4.5, 11.8 Hz, 1H), 2.55–2.38 (m, 3H), 2.36–2.06 (m, 4H), 1.59 (td, *J* = 3.7, 14.5 Hz, 1H), 1.48 (dd, *J* = 2.5, 12.3 Hz, 1H), 0.78–0.66 (m, 1H), 0.52–0.4 (m, 2H), 0.1–(−)0.02 (m, 2H); ^13^C NMR (100 MHz, CDCl_3_) *δ* 209.2, 171.3, 154.2, 143.4, 141.1, 138.9, 128.0, 124.8, 119.9, 118.3, 110.3, 105.3, 90.0, 82.7, 59.3, 55.5, 51.2, 43.8, 35.4, 33.7, 30.0, 26.9, 23.5, 20.0, 13.5, 9.4, 3.7. HRMS: calc. for C_27_H_30_NO_6_, 464.207313; found 464.2099. The corresponding HCl salt was obtained by using 1 M solution of HCl in Et_2_O. Anal (C_27_H_29_NO_6_·HCl·2H_2_O) C, H, N.

##### 14β-(3-(Furan-3-yl)propanoyl)-17-cyclopropylmethyl-7,8-dihydronoroxymorphinone (3c)

The product, free base, was obtained as a clear oil (78%). ^1^H NMR (400 MHz, CDCl_3_) *δ* 7.35 (t, *J* = 1.5 Hz, 1H), 7.30 (b s, 1H), 6.74 (d, *J* = 8.0 Hz, 1H), 6.58 (d, *J* = 8.0 Hz, 1H), 6.33 (s, 1H), 4.67 (s, 1H), 4.46 (d, *J* = 5.5 Hz, 1H), 3.08 (d, *J* = 18.3 Hz, 1H), 2.90–2.63 (m, 6H), 2.57–2.41 (m, 3H), 2.40–2.10 (m, 4H), 1.62 (td, *J* = 3.2, 13.8 Hz, 1H), 1.51 (dd, *J* = 2.5, 12.3 Hz, 1H), 0.98–0.83 (m, 1H), 0.78–0.66 (m, 1H), 0.55–0.40 (m, 2H), 0.14–0.00 (m, 2H); ^13^C NMR (100 MHz, CDCl_3_) *δ* 209.0, 171.9, 143.5, 143.0, 139.1, 138.8, 128.1, 125.1, 123.6, 120.0, 118.2, 110.8, 90.1, 82.7, 59.4, 55.6, 51.3, 43.9, 35.7, 35.5, 30.1, 27.0, 23.9, 20.1, 17.3, 9.5, 3.7. HRMS: calc. for C_27_H_30_NO_6_, 464.207313; found 464.2103. The corresponding HCl salt was obtained by using 1 M solution of HCl in Et_2_O. Anal (C_27_H_29_NO_6_·HCl·0.5H_2_O) C, H, N.

##### 14β-(3-(Thiophen-3-yl)propanoyl)-17-cyclopropylmethyl-7,8-dihydronoroxymorphinone (3d)

The product, free base, was obtained as a clear oil (72%). ^1^H NMR (400 MHz, CDCl_3_) *δ* 7.29–7.23 (m, 1H), 7.04 (b s, 1H), 7.01 (dd, *J* = 1.5, 5.0 Hz, 1H), 6.73 (d, *J* = 8.0 Hz, 1H), 6.58 (d, *J* = 8.0 Hz, 1H), 4.63 (s, 1H), 4.46 (d, *J* = 5.5 Hz, 1H), 3.12–3.00 (m, 3H), 2.90–2.74 (m, 3H), 2.69 (dd, *J* = 4.77, 11.8 Hz, 1H), 2.58–2.23 (m, 5H), 2.23–2.10 (m, 2H), 1.60 (td, *J* = 3.7, 14.3 Hz, 1H), 1.50 (d, *J* = 10.0 Hz, 1H), 0.80–0.66 (m, 1H), 0.57–0.41 (m, 2H), 0.16–0.01 (m, 2H); ^13^C NMR (100 MHz, CDCl_3_) *δ* 208.9, 171.2, 143.4, 140.9, 138.8, 127.8, 125.7, 124.9, 120.5, 119.9, 118.2, 90.0, 82.6, 59.4, 55.5, 51.2, 43.9, 36.0, 35.3, 30.0, 26.9, 25.6, 23.0, 20.9, 9.4, 3.7. HRMS: calc. for C_27_H_30_NO_5_S, 480.184469; found 480.1858. The corresponding HCl salt was obtained by using 1 M solution of HCl in Et_2_O. Anal (C_27_H_29_NO_5_S·HCl·2H_2_O) C, H, N.

##### 14β-(4,4-Dimethylpentanoyl)-17-cyclopropylmethyl-7,8-dihydronoroxymorphinone (3e)

The product, free base, was obtained as clear oil (30 mg, 37%). ^1^H NMR (500 MHz, CDCl_3_) *δ* 6.74 (d, *J* = 8.0 Hz, 1H), 6.60 (d, *J* = 8.0 Hz, 1H), 4.71 (s, 1H), 4.43 (d, *J* = 5.7 Hz, 1H), 3.08 (d, *J* = 18.6 Hz, 1H), 2.90–2.80 (m, 1H), 2.76–2.25 (m, 8H), 2.15 (td, *J* = 3.8, 12.0 Hz, 1H), 1.70–1.50 (m, 4H), 1.43–1.30 (m, 2H), 0.94 (s, 9H), 0.84–0.70 (m, 1H), 0.54–0.42 (m, 2H), 0.12–0.02 (m, 2H); ^13^C NMR (125 MHz, CDCl_3_) *δ* 209.2, 173.5, 143.4, 138.8, 129.0, 125.3, 120.0, 118.2, 90.2, 82.1, 59.5, 55.8, 51.4, 43.8, 38.8, 35.8, 31.2, 29.1, 28.0, 23.9, 23.1, 9.50, 3.9, 3.7. HRMS: calc. for C_27_H_36_NO_5_, 454.259348; found 454.2614. The corresponding HCl salt was obtained by using 1 M solution of HCl in Et_2_O. Anal (C_27_H_35_NO_5_·HCl·0.5H_2_O) C, H, N.

##### 14β-(3-Phenylpropanoyl)-17-cyclopropylmethyl-4,5-epoxy-3,14-dihydroxymorphinan (11a)

Isolated as a white solid (92 mg, 94%). *R*_f_ 0.10, (at 30% EtOAc/hexane, 0.5% NH_3_), ^1^H NMR (400 MHz, CDCl_3_) *δ* 7.15–7.25 (m, 5H), 6.63–6.65 (d, *J* = 8.0 Hz, 2H), 6.49–6.51 (d, *J* = 8.0 Hz, 2H), 4.62 (t, *J* = 5.5 Hz, 1H), 4.28–4.29 (d, *J* = 4.0 Hz, 1H), 3.69–3.72 (t, *J* = 6.0 Hz, 2H), 2.97–3.01 (d, *J* = 20.0 Hz, 1H), 2.94–2.97 (m, 2H), 2.62–2.71 (m, 3H), 2.27–2.260 (m, 2H), 2.23–2.26 (m, 3H), 2.20–2.23 (m, 1H), 1.99–2.12 (m, 2H), 1.79–1.82 (t, *J* = 14.5 Hz, 1H), 1.26–1.32 (d, *J* = 8.0, 1H), 1.22–1.26 (t, *J* = 9.7 Hz, 1H), 0.56–0.60 (m, 1H), 0.39–0.41 (d, *J* = 8.0 Hz, 2H), −0.02–0.02 (d, *J* = 8.0 Hz, 2H); ^13^C NMR (100.6 MHz, CDCl_3_) *δ* 170.6, 147.0, 140.3, 133.4, 132.8, 131.1, 128.5, 128.4, 126.4, 122.0, 118.4, 90.2, 70.5, 62.6, 59.3, 56.7, 44.3, 35.6, 31.0, 30.7, 28.9, 23.1, 17.2, 9.5, 3.9, 3.8; HRMS: calc. for C_29_H_33_NO_4_, 450.2410; found 450.2378; anal (C_29_H_33_NO_4_·HCl·0.5H_2_O) C, H, N.

##### 14β-(3-(Furan-2-yl)propanoyl)-17-cyclopropylmethyl-4,5-epoxy-3,14-dihydroxymorphinan (11b)

The product, free base, was obtained as white foam (100%). ^1^H NMR (400 MHz, MeOD) *δ* 7.39 (s, 1H), 6.76 (d, *J* = 8.2 Hz, 1H), 6.71 (d, *J* = 8.2 Hz, 1H), 6.32 (dd, *J* = 1.76, 3.0 Hz, 1H), 6.14 (d, *J* = 3.3 Hz, 1H), 5.32 (d, *J* = 6.0 Hz, 1H), 4.77 (t, *J* = 7.3 Hz, 1H), 3.47–3.34 (m, 2H), 3.28–3.18 (m, 2H), 3.15–2.89 (m, 5H), 2.84 (td, *J* = 4.3, 13.0 Hz, 1H), 2.56 (td, *J* = 4.3, 13.0 Hz, 1H), 2.50–2.40 (m, 1H), 2.18–2.08 (m, 1H), 1.71 (dd, *J* = 3.5, 14.0 Hz, 1H), 1.44–1.25 (m, 4H), 1.10–0.98 (m, 1H), 0.87–0.77 (m, 1H), 0.76–0.68 (m, 1H), 0.56–0.46 (m, 2H); ^13^C NMR (100 MHz, MeOD) *δ* 174.4, 155.5, 144.3, 143.2, 142.7, 130.3, 122.1, 120.7, 119.8, 111.5, 106.8, 88.5, 84.8, 59.6, 59.1, 47.1, 35.1, 29.5, 29.0, 26.8, 25.3, 24.2, 17.4, 6.9, 6.4, 3.5. HRMS: calc. for C_27_H_32_NO_5_, 450.2280; found 450.2378. Anal (C_27_H_31_NO_5_·HCl·0.5H_2_O) C, H, N.

##### 14β-(3-(Furan-3-yl)propanoyl)-17-cyclopropylmethyl-4,5-epoxy-3,14-dihydroxymorphinan (11c)

The product, free base, was obtained as clear oil (93%). ^1^H NMR (400 MHz, MeOD) *δ* 7.41 (s, 1H), 7.37 (s, 1H), 6.75 (d, *J* = 8.0 Hz, 1H), 6.71 (d, *J* = 8.0 Hz, 1H), 6.40 (s, 1H), 5.32 (d, *J* = 5.5 Hz, 1H), 4.78–4.70 (m, 1H), 3.50–3.34 (m, 2H), 3.28–3.18 (m, 2H), 3.15–2.95 (m, 2H), 2.90–2.72 (m, 4H), 2.67–2.53 (m, 1H), 2.52–2.40 (m, 1H), 2.18–2.05 (m, 1H), 1.66 (d, *J* = 11.0 Hz, 1H), 1.44–1.22 (m, 4H), 1.13–1.00 (m, 1H), 0.87–0.76 (m, 1H), 0.75–0.65 (m, 1H), 0.64–0.55 (m, 1H), 0.55–0.45 (m, 1H); ^13^C NMR (100 MHz, MeOD) *δ*174.8, 144.3, 143.1, 140.7, 130.3, 125.1, 122.1, 120.7, 120.0, 112.1, 88.5, 84.5, 59.5, 58.9, 47.1, 37.4, 29.5, 28.9, 26.8, 25.4, 21.1, 17.4, 6.9, 6.5, 3.6. HRMS: calc. for C_27_H_32_NO_5_, 450.2280; found 450.2339. Anal (C_27_H_31_NO_5_·HCl·H_2_O) C, H, N.

##### 14β-(3-(Thiophen-2-yl)propanoyl)-17-cyclopropylmethyl-4,5-epoxy-3,14-dihydroxymorphinan (11d)

The product, free base, was obtained as clear oil (88%). ^1^H NMR (400 MHz, CDCl_3_) *δ* 7.30–7.24 (m, 1H), 7.08–6.98 (m, 2H), 6.72 (d, *J* = 8.0 Hz, 1H), 6.57 (d, *J* = 8.0 Hz, 1H), 4.71 (t, *J* = 6.8 Hz, 1H), 4.38 (d, *J* = 5.3 Hz, 1H), 3.11–2.99 (m, 3H), 2.84–2.62 (m, 3H), 2.52 (dd, *J* = 5.5, 18.3 Hz, 1H), 2.43 (d, *J* = 14.0 Hz, 1H), 2.38–2.22 (m, 3H), 2.14 (td, *J* = 3.7, 12.0 Hz, 1H), 2.10–2.00 (m, 1H), 1.47–1.20 (m, 5H), 0.8–0.67 (m, 1H), 0.55–0.4 (m, 2H), 0.14–0.0 (m, 2H); ^13^C NMR (100 MHz, CDCl_3_) *δ* 171.7, 142.1, 141.1, 139.4, 130.8, 128.0, 125.9, 125.4, 120.4, 118.7, 116.8, 89.2, 84.0, 59.5, 56.0, 47.0, 44.6, 36.2, 30.2, 28.5, 26.2, 25.6, 23.1, 16.8, 9.5, 3.8, 3.7. C_27_H_32_NO_4_S, 466.2052; found 466.2163. The corresponding HCl salt was obtained by using 1 M solution of HCl in Et_2_O. Anal (C_27_H_31_NO_4_S·HCl·0.5H_2_O) C, H, N.

##### 14β-(4,4-Dimethylpentanoyl)-17-cyclopropylmethyl-4,5-epoxy-3,14-dihydroxymorphinan (11e)

The product, free base, was obtained as clear oil (94%). ^1^H NMR (400 MHz, CDCl_3_) *δ* 6.71 (d, *J* = 8.0 Hz, 1H), 6.55 (d, *J* = 8.0 Hz, 1H), 4.74 (t, *J* = 7.5 Hz, 1H), 4.31 (d, *J* = 5.3 Hz, 1H), 3.05 (d, *J* =18.3 Hz, 1H), 2.67 (dd, *J* = 4.5, 11.8 Hz, 1H), 2.55–2.40 (m, 2H), 2.39–2.24 (m, 5H), 2.17–2.06 (m, 2H), 1.60 (dd, *J* = 6.8, 8.0 Hz, 2H), 1.56–1.46 (m, 1H), 1.44–1.30 (m, 3H), 1.29–1.19 (m, 1H), 0.92 (s, 9H), 0.8–0.7 (m, 1H), 0.5–0.4 (m, 2H), 0.11–0.0 (m, 2H); ^13^C NMR (100 MHz, CDCl_3_) *δ* 173.3, 142.1, 139.4, 130.9, 126.0, 118.7, 116.8, 89.2, 83.4, 59.6, 56.2, 47.1, 44.5, 38.8, 31.4, 30.2, 30.0, 29.1, 28.5, 26.3, 23.2, 17.0, 9.5, 3.9, 3.5. C_27_H_38_NO_4_, 440.2801; found 440.2899. The corresponding HCl salt was obtained by using 1 M solution of HCl in Et_2_O. Anal (C_27_H_37_NO_4_·HCl·1.5H_2_O).

### Cell lines and cell culture

Human (h) DOP- and hNOP-receptor CHO cell lines were generous gifts from Larry Toll at the Torrey Pines Institute. The hMOP- and hKOP-CHO cell lines were generous gifts from John M. Streicher at the University of Arizona. All cells were cultured in 50 : 50 DMEM/F12 media with 10% heat-inactivated FBS and 1× penicillin/streptomycin supplement (all Gibco brand) in a 37 °C humidified incubator with 5% CO_2_ atmosphere. Propagation cultures were further maintained with 500 μg mL^−1^ G418. Cultures were propagated for no more than 30 passages. Cell pellets for experiments were prepared by growth in 15 cm^2^ plates, harvested with 5 mM EDTA in 50 mM Tris HCl pH 7.4. Cell pellets were resuspended in 50 mM Tris HCl pH 7.4 and homogenized with a tissue grinder for 15–30 seconds on ice. Crude membranes were spun down at 15 000*g* for 30 min, washed once followed by recentrifugation. Membrane preparations were stored at −80 °C prior to use.

### Competition radioligand binding

hDOP-, hKOP- or hMOP-receptor containing CHO membrane preparations were diluted to 10–20 μg in 50 mM Tris-HCl buffer (pH 7.4) and incubated with a fixed concentration of ^3^H-diprenorphine (0.2–0.4 nM) and varying concentrations of test compound. With hNOP receptor-CHO cells, ^3^H-nociceptin was used in an analogous manner. Reactions were performed in 400 μL volumes in 96 well plates and incubated at 37 °C for 3 hours. Reactions were terminated by rapid filtration through 24 well format GF/B filter mat (PerkinElmer) and washed with cold 25 mM Tris HCl pH 7.4 buffer, dried, and ECOLUME™ scintillation cocktail from MP Biomedicals (Santa Cruz, CA, USA) was added. Radioactivity retained on the filters was determined using a MicroBeta2 96-well format 6 detector scintillation counter (PerkinElmer). *K*_i_ values were calculated from the inhibitory concentration_50_ (IC_50_) of each competitor ligand using GraphPad Prism (v7.0).

### ^35^S-GTPγS binding

Briefly, hDOP, hKOP, hMOP- or hNOP-receptor containing CHO membranes were combined with test compound, 50 pM [^35^S]GTPγS (Perkin Elmer), and 10–15 μg membrane homogenate in GTPγS assay buffer (50 mM Tris HCl pH 7.4, 125 mM NaCl, 5 mM MgCl_2_, 1 mM EDTA and 30 μM GDP). The 200 μL reaction was incubated at 30 °C for 50 minutes in 96 well plates, then collected by filtration and measured for reactivity as described for the binding experiments. Potency (EC_50_) and *E*_MAX_ values were calculated using the three-variable log(agonist) *vs.* response curve in GraphPad Prism 7.0.

## Conclusions

Naltrexone analogues with C_14_-ester side chains have dramatically improved NOP partial agonist affinity/potency coupled with partial agonist activity at MOP receptors. Potency at NOP is further increased through removal of the C_6_-carbonyl. The mixed MOP:NOP partial agonist profile is retained across these series with the major differences between analogues being in the MOP:NOP selectivity. These series offer promising candidates for improved analgesics and potentially, addiction treatments and will allow for future studies to determine the optimal profile for these indications.

## Author contributions

MO, KO and GC-K performed the experiments and analyzed data. SMH, SBK, LJ performed the computational studies. SMH and JRT devised the study and with KO wrote the manuscript.

## Conflicts of interest

SMH is an inventor on the patent that describes these compounds.

## Supplementary Material

MD-OLF-D5MD00685F-s001

## Data Availability

Chemical characterisation data supporting this article have been uploaded as part of the supplementary information (SI). Supplementary information: ^1^H and ^13^C NMR spectra and image from docking of 11e to MOP. See DOI: https://doi.org/10.1039/d5md00685f
