# Supplementary material for: Synthesis and evaluation of 14β-acyl substituted 17-cyclopropylmethyl-7,8-dihydromorphinone derivatives: mixed partial agonists at mu opioid and nociception/orphanin FQ peptide receptors
Source: RSC Med Chem. 2026 Feb 12;17(4):1950–60. doi: 10.1039/d5md00685f (PMC12951310; doi:10.1039/d5md00685f)
Supplement: MD-017-D5MD00685F-s001 [file MD-017-D5MD00685F-s001.pdf]

Supplementary Information (SI) for RSC Medicinal Chemistry.
This journal is © The Royal Society of Chemistry 2026

Supplementary Information

Synthesis and evaluation of 14β-acyl substituted 17-cyclopropylmethyl-7,8-dihydromorphinone derivatives: mixed partial agonists at mu opioid and nociception/orphanin FQ peptide receptors

Mehrnoosh Ostovar[a], Keith Olsen[b], Gerta Cami-Kobeci[a], John R. Traynor[b,c],Luka Jeramaz[d], Stewart B. Kirton[e] Stephen M. Husbands[a*]

[a.] Medicinal Chemistry Section, Department of Life Sciences, University of Bath, Bath, BA2 7AY, United Kingdom
[b.] Department of Pharmacology and Edward F Domino Research Center, University of Michigan, Ann Arbor, MI 48109 USA
[c.] Department of Medicinal Chemistry, University of Michigan, Ann Arbor, MI 48109 USA
[d.] School of Health, Medicine and Life Sciences, University of Hertfordshire, Hatfield, Herts, AL10 9AB, United Kingdom
[e.] School of Applied and Health Science, London South Bank University, London, SE1 0AA, United Kingdom
.

S2 – S11: ^{1}H and ^{13}C NMR of final compounds

S12: Figure showing structures of **11e** β-FNA in the MOP binding pocket

3a

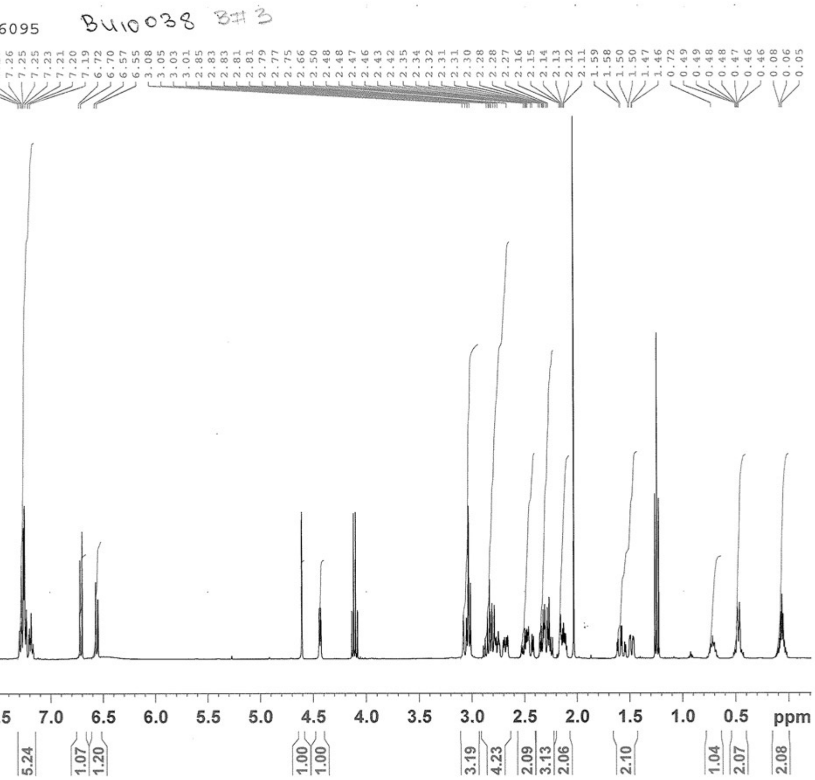

BRUKER

NAME	Mar08-2016-GC940
EXPNO	10
PROCNO	1
Date_	20160308
Time	10.54
INSTRUM	AVIII400
PROBHD	5 mm PABBO BB-
PULPROG	zg30
TD	65536
SOLVENT	CDCl3
NS	16
DS	2
SWH	8223.685 Hz
FIDRES	0.125483 Hz
AQ	3.9846387 sec
RG	32
DW	60.800 usec
DE	17.48 usec
TE	298.2 K
D1	1.00000000 sec
TD0	1

======== CHANNEL f1 ========

NUC1	1H
P1	11.90 usec
PL1	-1.00 dB
PL1W	12.26963711 W
SFO1	400.0424704 MHz
SI	65536
SF	400.0399837 MHz
WDW	EM
SSB	0
LB	0.20 Hz
GB	0
PC	1.00

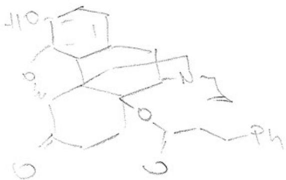

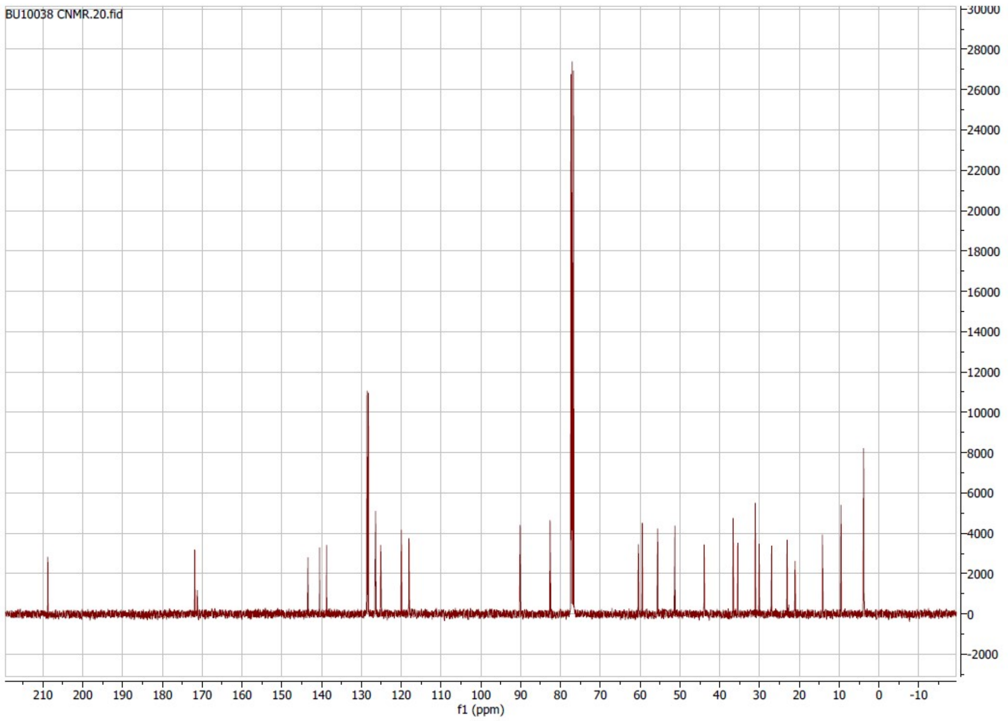
BU10038 CNMR.20.fid
f1 (ppm)

3b

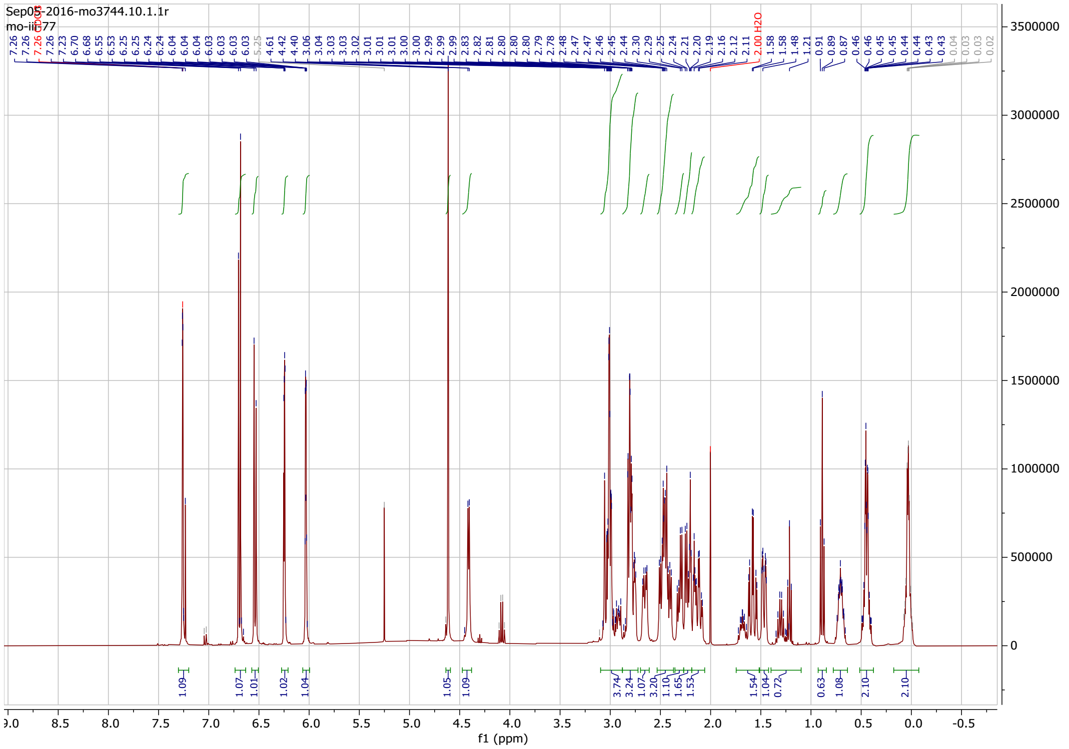

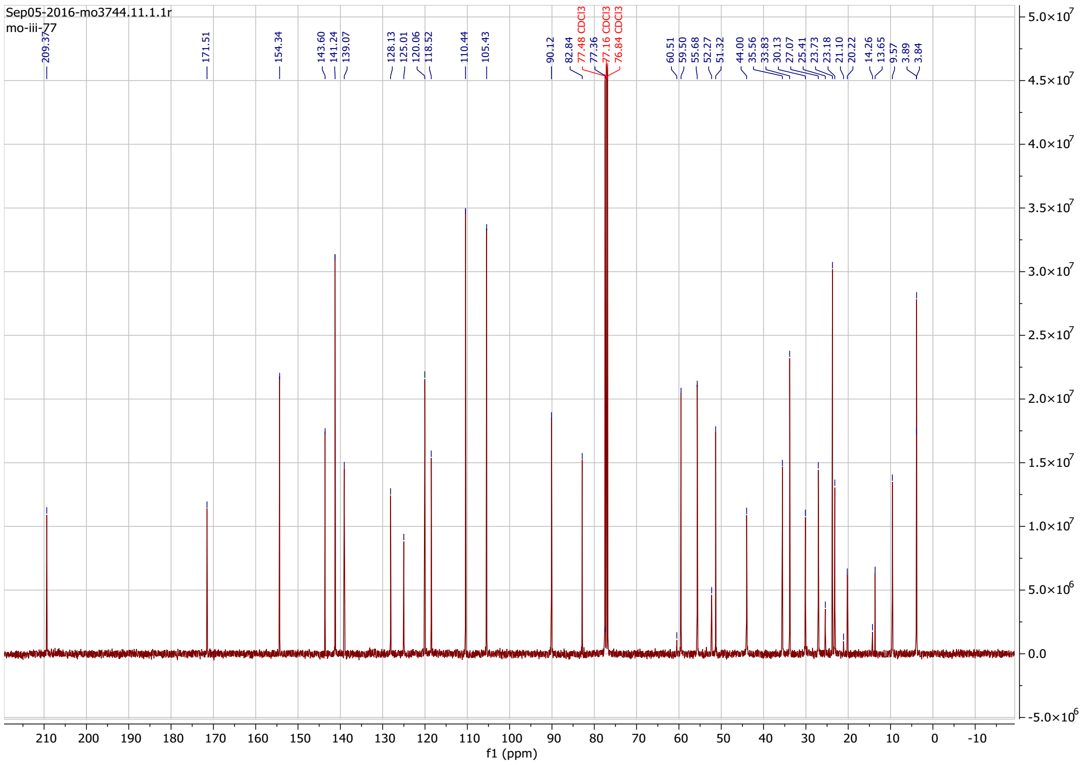

3c

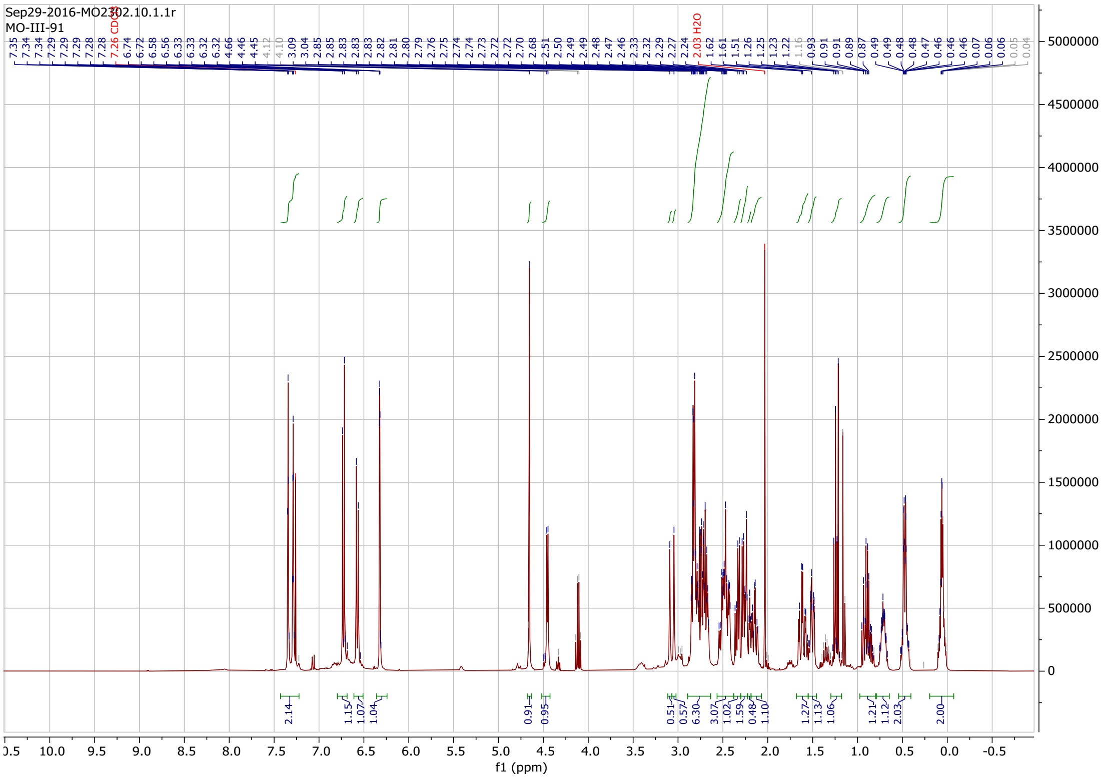
Sep29-2016-MO2302.10.1.1r
MO-III-91
f1 (ppm)

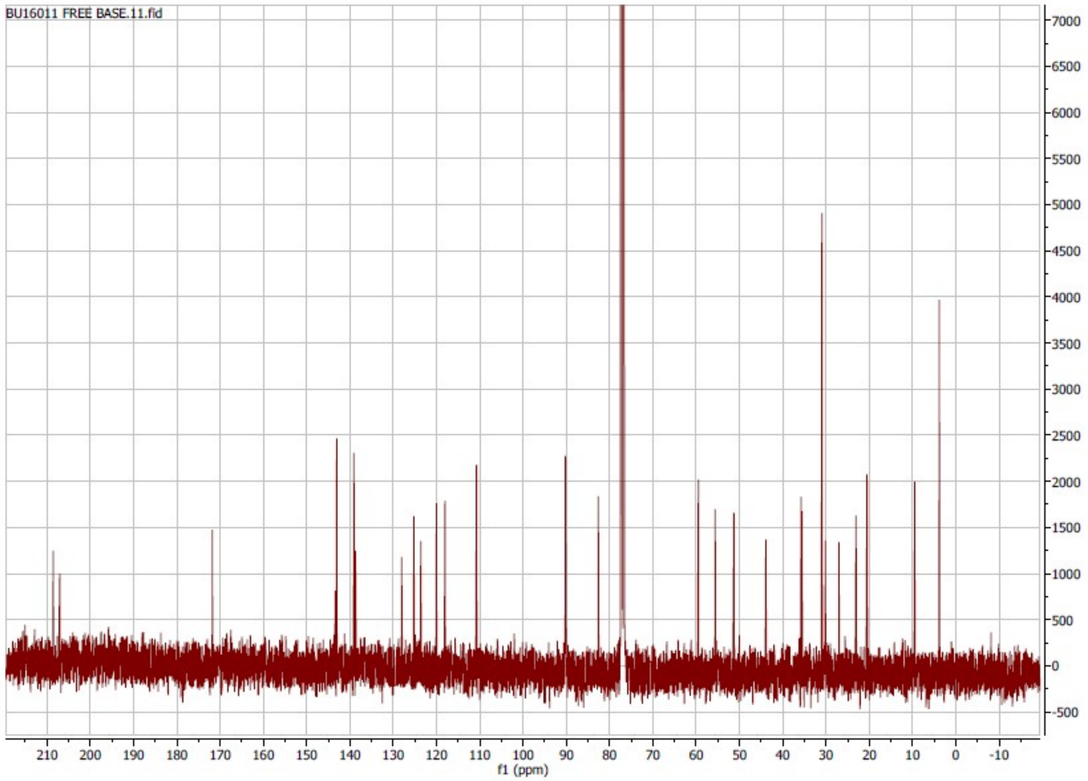
BU16011 FREE BASE.11.fid
f1 (ppm)

3d

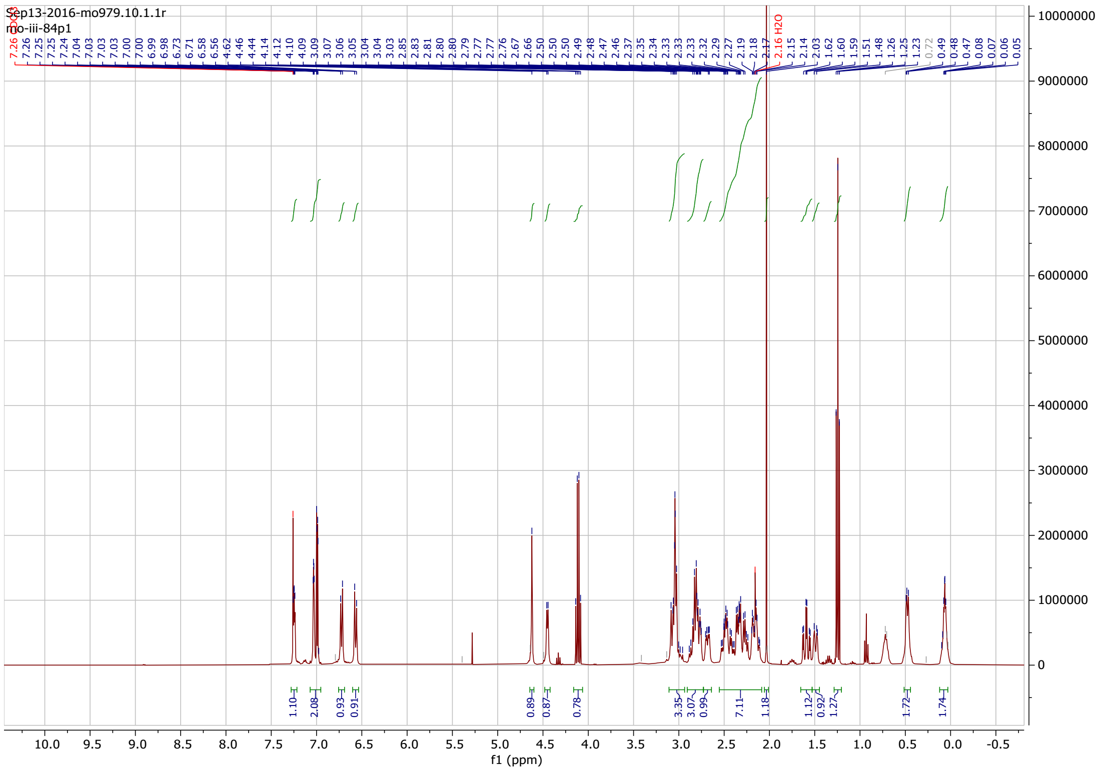

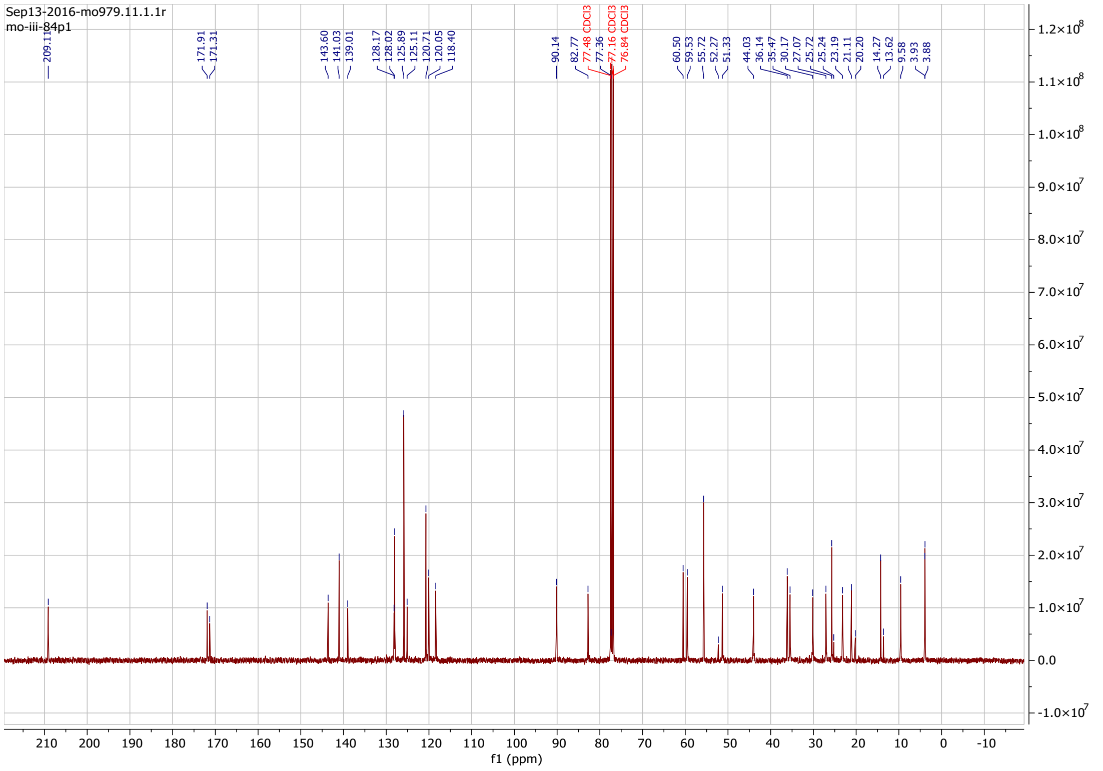

3e

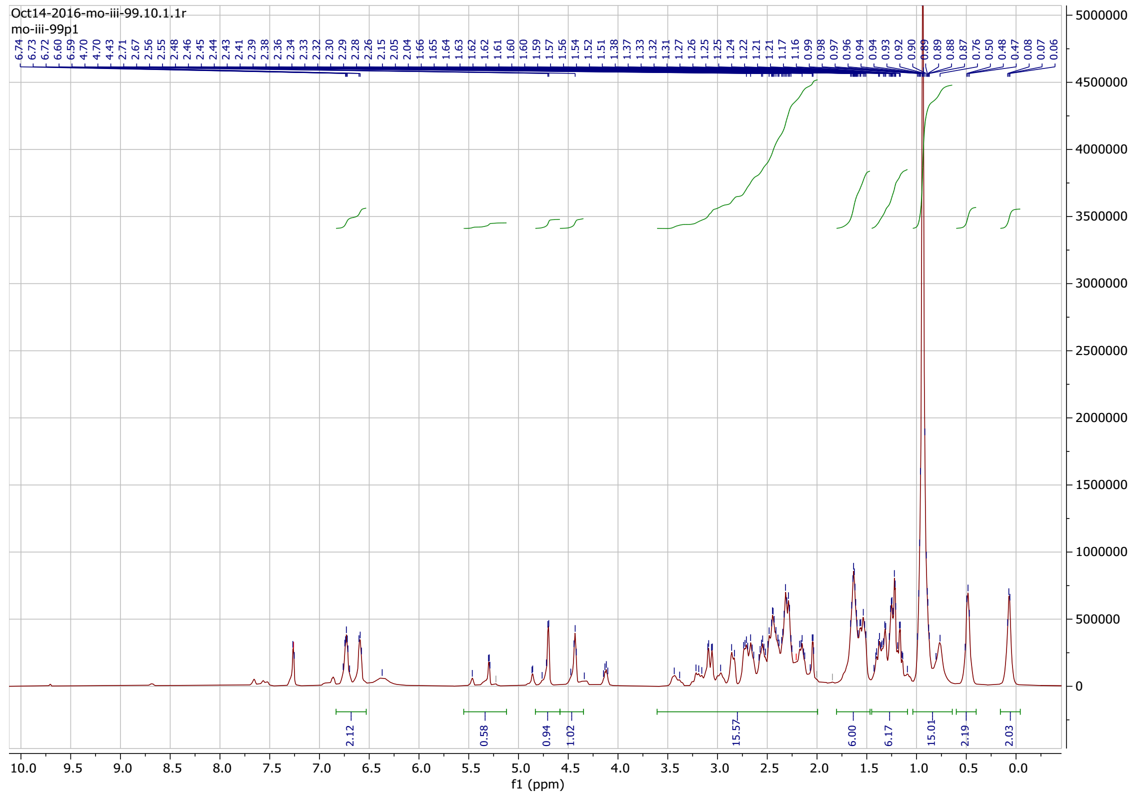
Oct14-2016-mo-iii-99.10.1.1r
mo-iii-99p1
6.74 6.73 6.72 6.60 6.59 4.70 4.70 4.43 2.71 2.67 2.56 2.55 2.48 2.46 2.45 2.44 2.43 2.41 2.39 2.38 2.36 2.34 2.33 2.32 2.30 2.29 2.28 2.26 2.15 2.05 2.04 1.66 1.65 1.64 1.63 1.62 1.62 1.61 1.60 1.60 1.59 1.57 1.56 1.54 1.52 1.51 1.38 1.37 1.33 1.32 1.31 1.27 1.26 1.25 1.25 1.24 1.22 1.21 1.21 1.17 1.16 0.99 0.98 0.97 0.96 0.94 0.94 0.93 0.92 0.90 0.89 0.89 0.88 0.87 0.76 0.50 0.48 0.47 0.08 0.07 0.06
2.12 0.58 0.94 1.02 15.57 6.00 6.17 15.01 2.19 2.03
f1 (ppm)

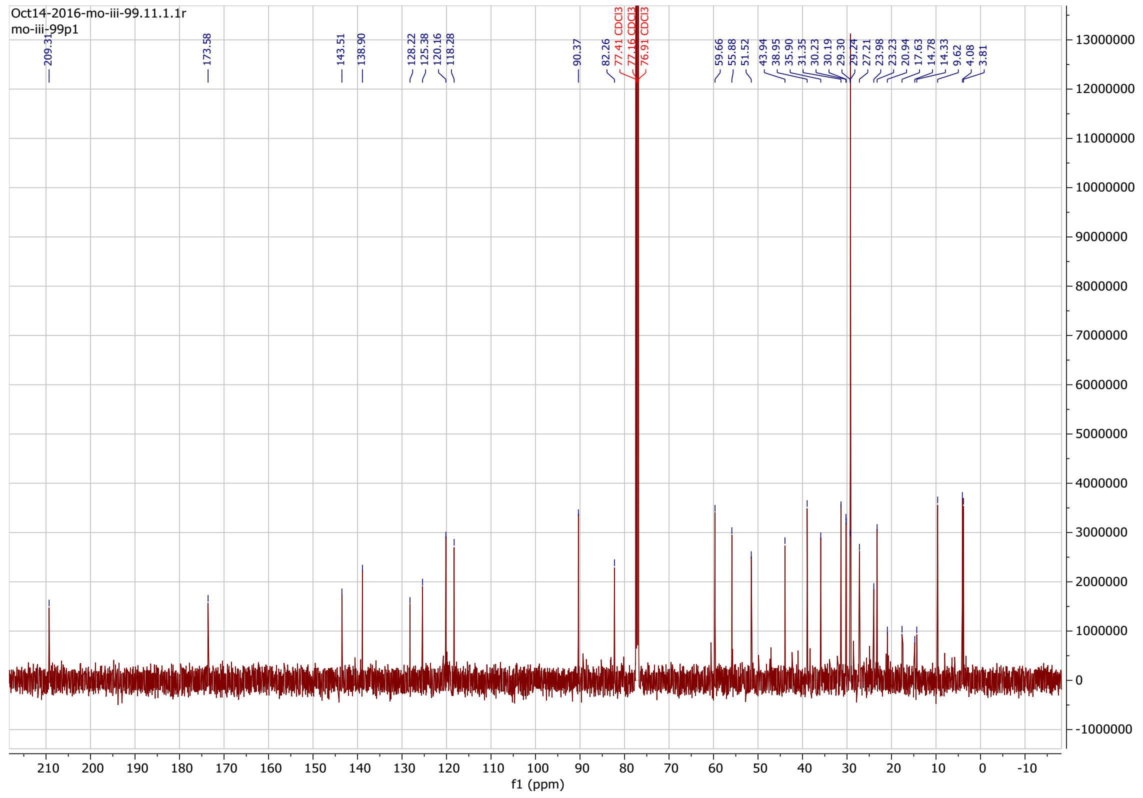
Oct14-2016-mo-iii-99.11.1.1r
mo-iii-99p1
209.31 173.58 143.51 138.90 128.22 125.38 120.16 118.28 90.37 82.26 77.41 CDCl3 77.16 CDCl3 76.91 CDCl3 59.66 55.88 51.52 43.94 38.95 35.90 31.35 30.23 30.19 29.30 29.24 27.21 23.98 23.23 20.94 17.63 14.78 14.33 9.62 4.08 3.81
f1 (ppm)

11a

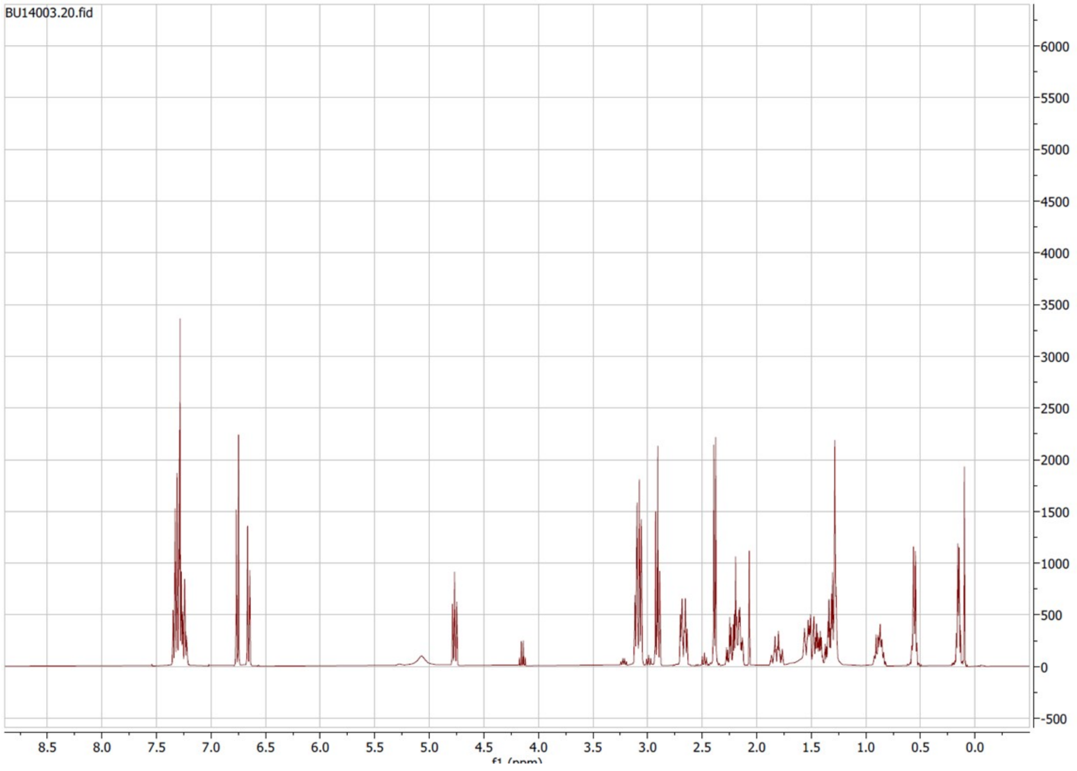

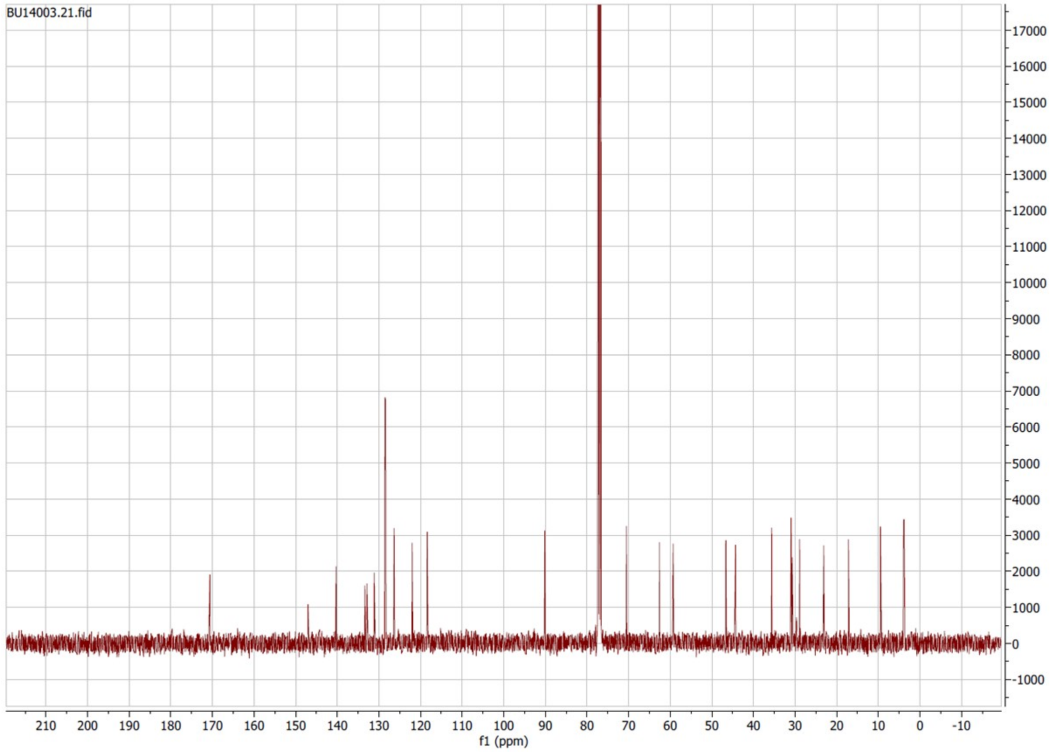

11b

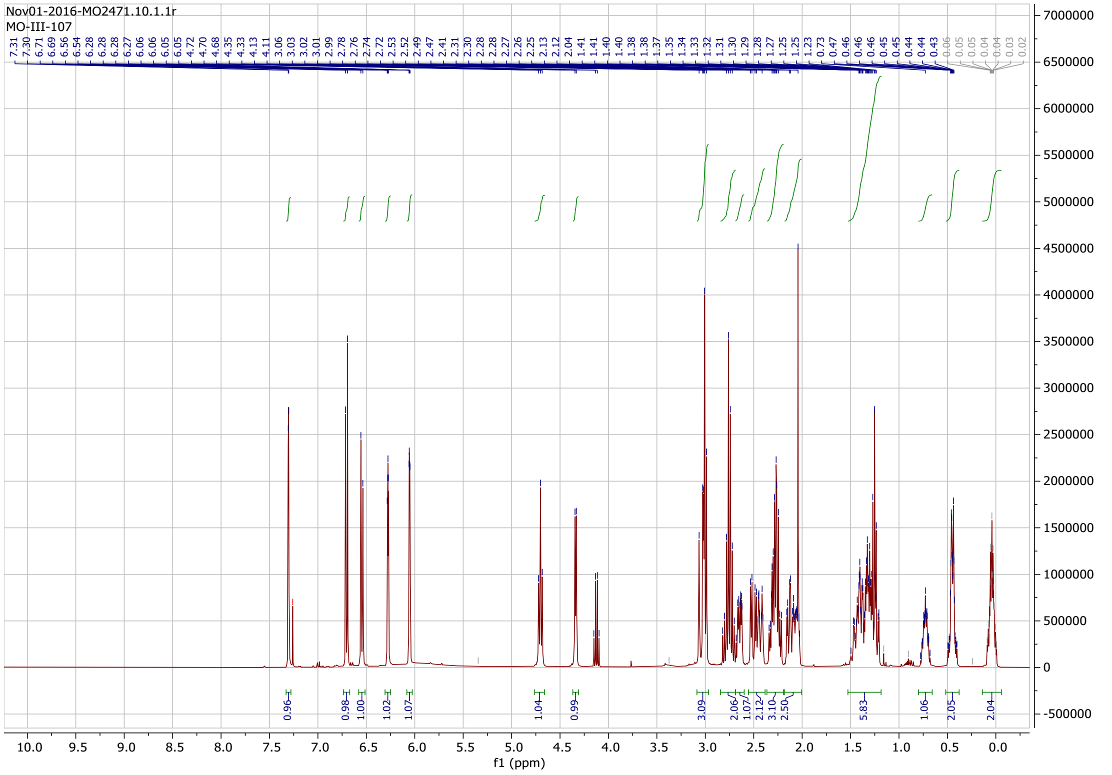

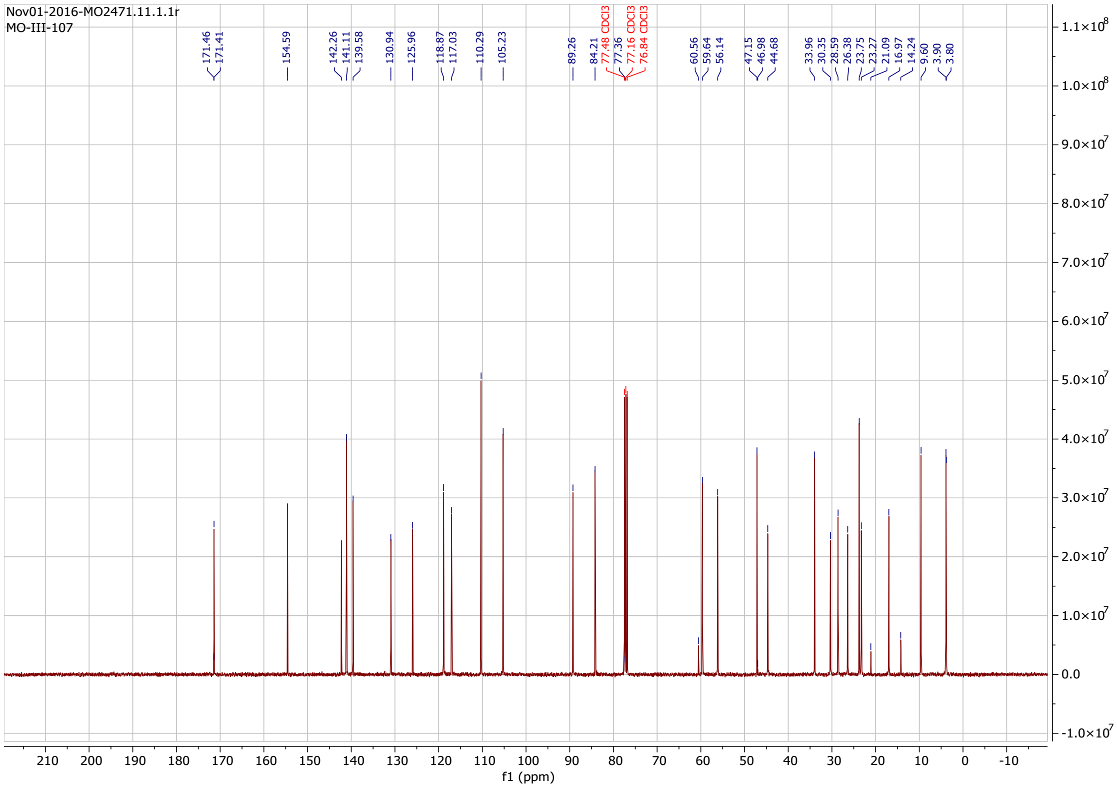

11c

This report was created by ACD/NMR Processor Academic Edition. For more information go to www.acdlabs.com/nmrproc/

16/06/2017 12 37 04

**Acquisition Time (sec)**	3 9846	**Comment**	mo-iii-115	**Date**	05 Jun 2017 17 55 28		
**Date Stamp**	05 Jun 2017 17 55 28						
**File Name**	\\campus\Files\Faculty of Science\Facilities-Temp\CCAF\Archive\9West\nmr-02\Jun05-2017-mo84\10\fid					**Frequency (MHz)**	400 08
**Nucleus**	1H	**Number of Transients**	16	**Origin**	AVIII400	**Original Points Count**	32768
**Owner**	Administrator	**Points Count**	32768	**Pulse Sequence**	zg30	**Receiver Gain**	101 00
**SW(cyclical) (Hz)**	8223 68	**Solvent**	METHANOL-d4	**Spectrum Offset (Hz)**	2461 0195	**Spectrum Type**	STANDARD
**Sweep Width (Hz)**	8223 43	**Temperature (degree C)**	25 160				

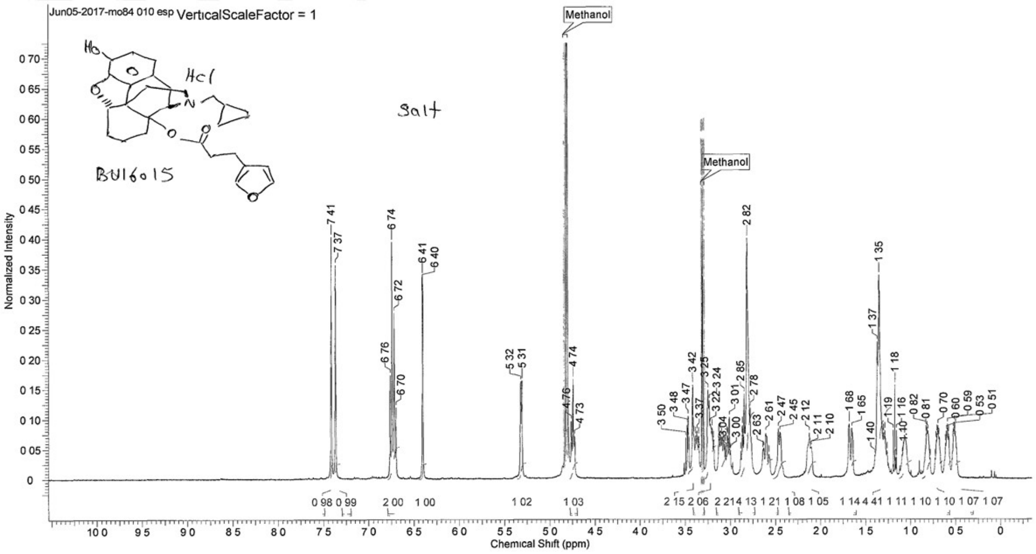

This report was created by ACD/NMR Processor Academic Edition. For more information go to www.acdlabs.com/nmrproc/

16/06/2017 12 40 05

Acquisition Time (sec)	1 3631	***Comment***	mo-III-115	***Date***	05 Jun 2017 23 04 48		
Date Stamp	05 Jun 2017 23 04 48						
File Name	\\campus\Files\Faculty of Science\Facilities-Temp\CCAF\Archive\9West\nmr-02\Jun05-2017-mo84\11\fid					***Frequency (MHz)***	100 60
Nucleus	13C	***Number of Transients***	2000	***Origin***	AVIII400	***Original Points Count***	32768
Owner	Administrator	***Points Count***	32768	***Pulse Sequence***	zgpg30	***Receiver Gain***	1620 00
SW(cyclical) (Hz)	24038 46	***Solvent***	METHANOL-d4	***Spectrum Offset (Hz)***	10232 6172	***Spectrum Type***	STANDARD
Sweep Width (Hz)	24037 73	***Temperature (degree C)***	25 160				

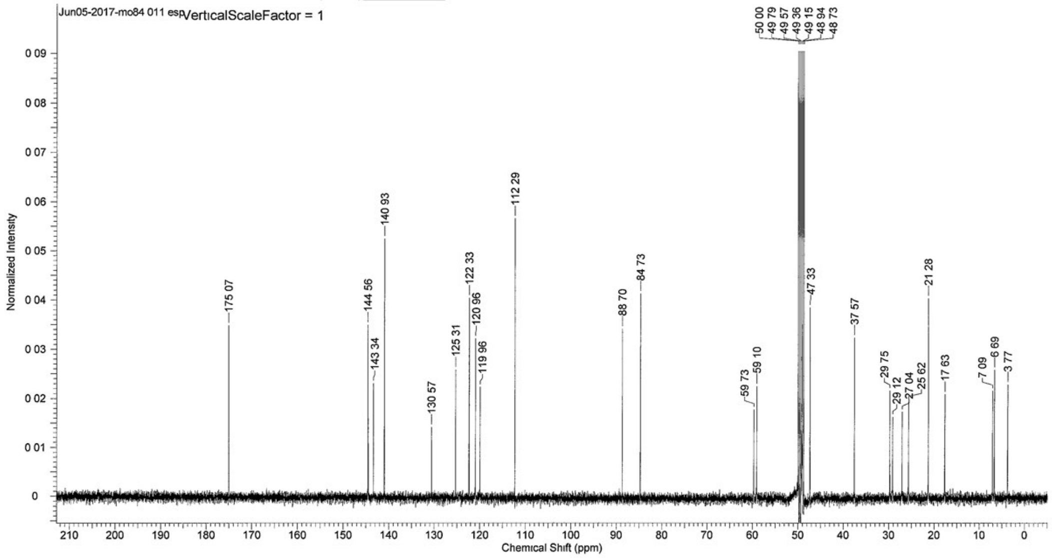

11d

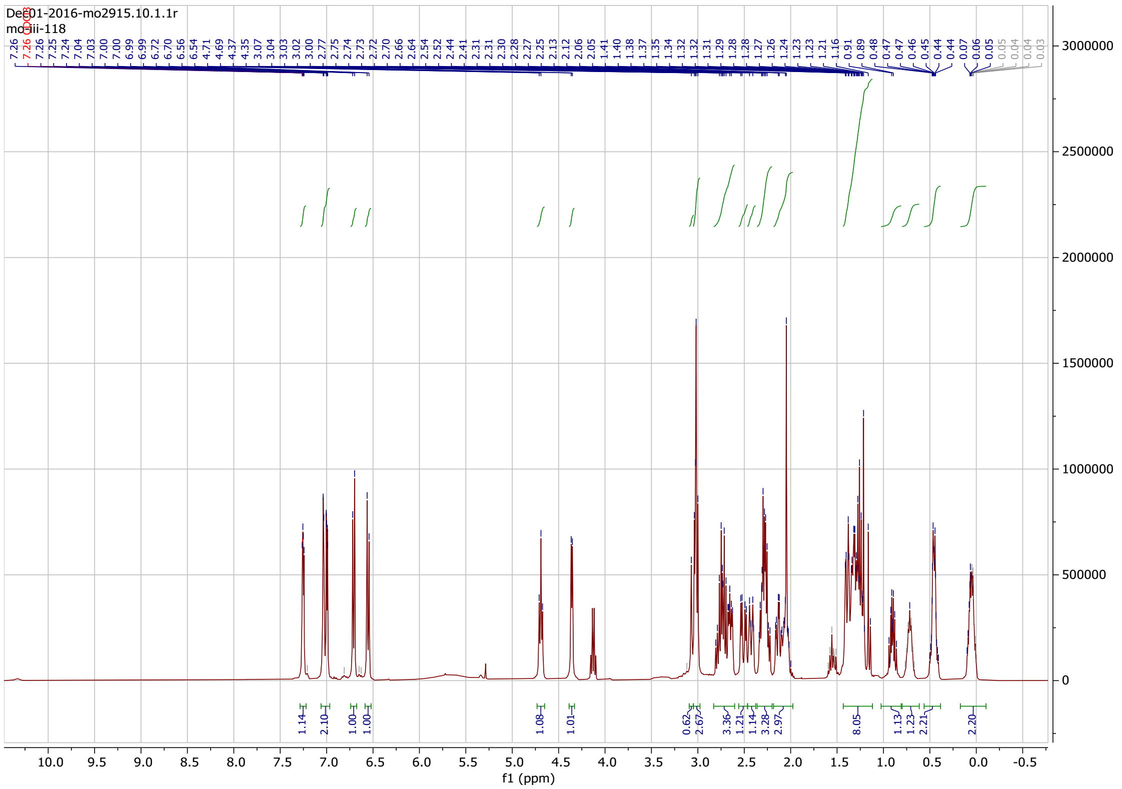

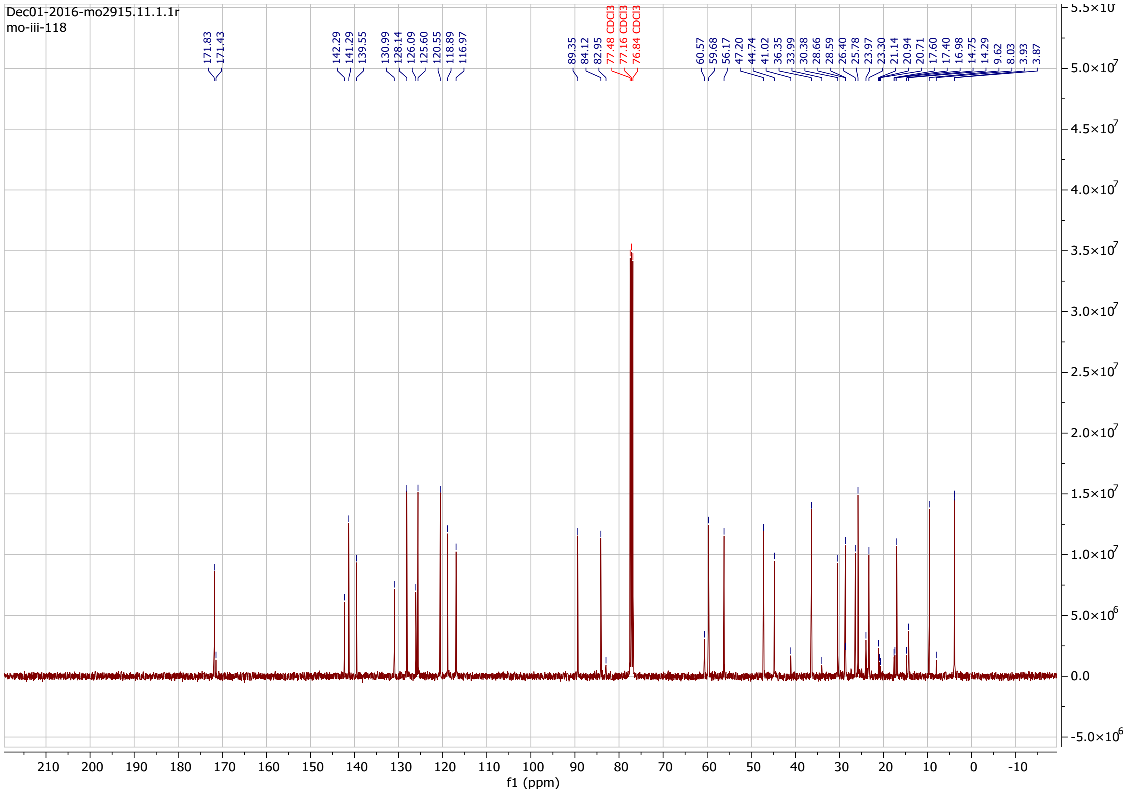

11e

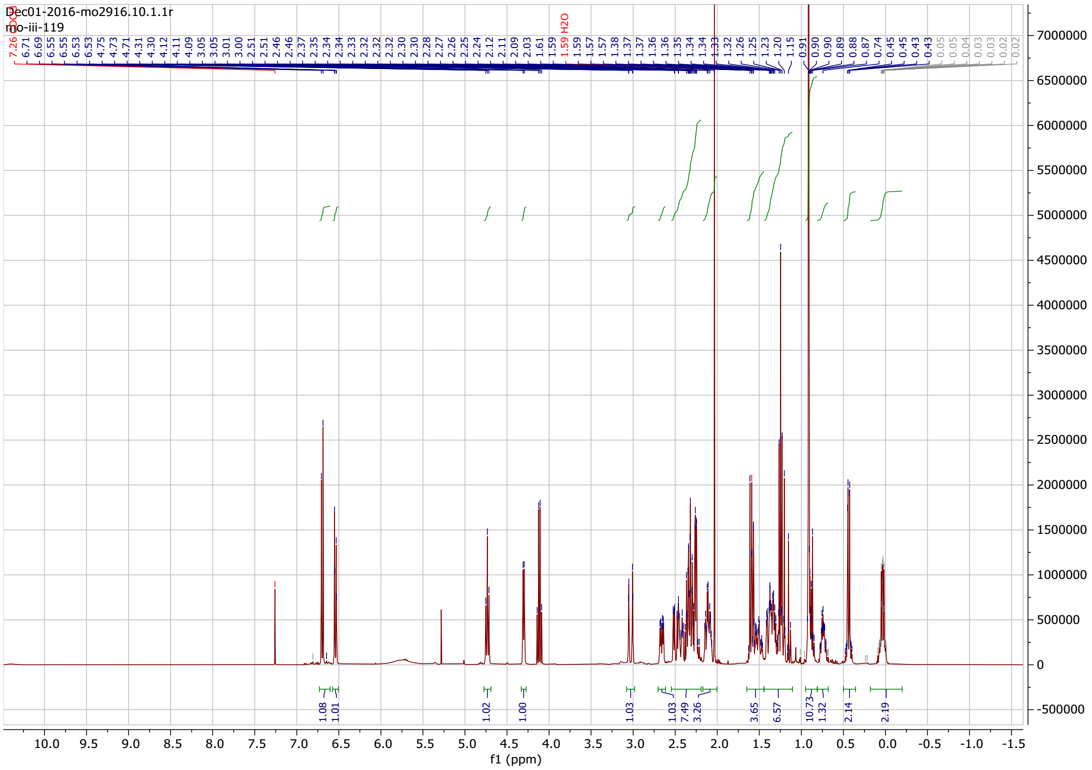
Dec01-2016-mo2916.10.1.1r
mo-iii-119
f1 (ppm)

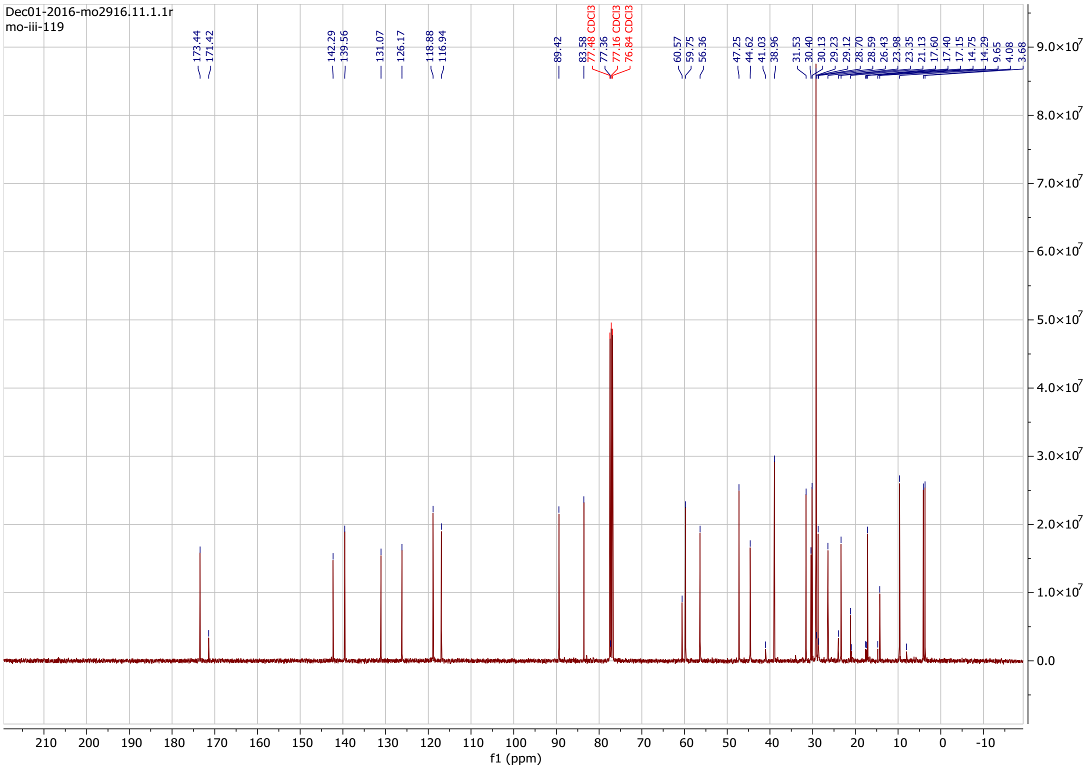
Dec01-2016-mo2916.11.1.1r
mo-iii-119
f1 (ppm)

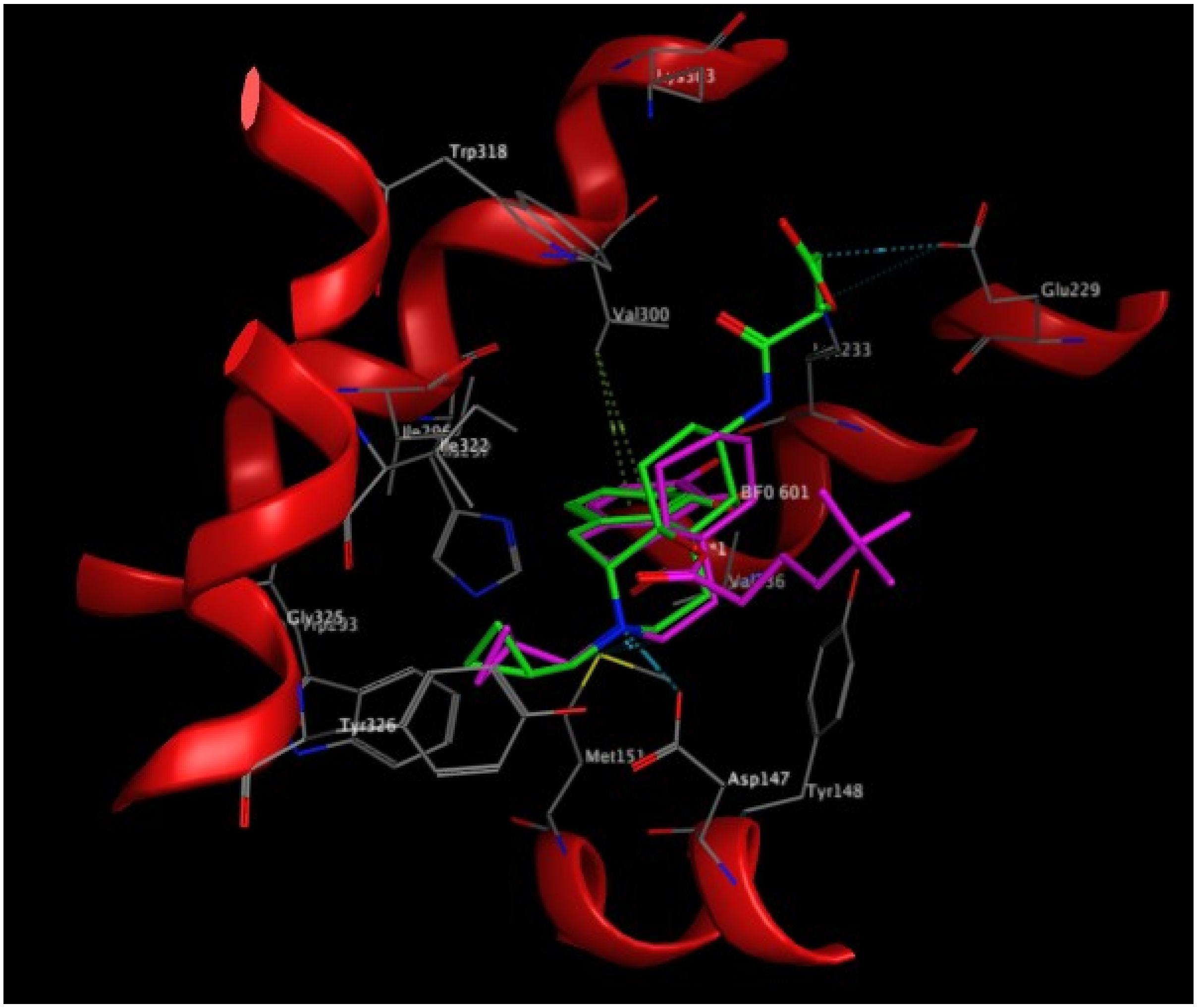

Structures of 11e (purple) and the MOP co-crystallized ligand, β-FNA (green) in the MOP binding pocket
